# Development and Use of a Monoclonal Antibody Specific for the *Candida albicans* Cell-Surface Protein Hwp1

**DOI:** 10.3389/fcimb.2022.907453

**Published:** 2022-06-27

**Authors:** Soon-Hwan Oh, Hélène Martin-Yken, David A. Coleman, Etienne Dague, Lois L. Hoyer

**Affiliations:** ^1^ Department of Pathobiology, University of Illinois at Urbana-Champaign, Urbana, IL, United States; ^2^ Toulouse Biotechnology Institute, Université de Toulouse, CNRS, INRAE, INSA, Toulouse, France; ^3^ LAAS-CNRS, Université de Toulouse, CNRS, Toulouse, France

**Keywords:** *Candida albicans*, adhesion, cell-surface protein, monoclonal antibody, Hwp1, biofilm formation

## Abstract

The *Candida albicans* cell-surface protein Hwp1 functions in adhesion to the host and in biofilm formation. A peptide from the Gln-Pro-rich adhesive domain of Hwp1 was used to raise monoclonal antibody (MAb) 2-E8. MAb 2-E8 specificity for Hwp1 was demonstrated using a *hwp1/hwp1 C. albicans* isolate and strains that expressed at least one *HWP1* allele. Immunofluorescence and atomic force microscopy experiments using MAb 2-E8 confirmed *C. albicans* germ-tube-specific detection of the Hwp1 protein. MAb 2-E8 also immunolabeled the tips of some *Candida dubliniensis* germ tubes grown under conditions that maximized *HWP1* expression. The phylogeny of *HWP1* and closely related genes suggested that the Gln-Pro-rich adhesive domain was unique to *C. albicans* and *C. dubliniensis* focusing the utility of MAb 2-E8 on these species. This new reagent can be used to address unanswered questions about Hwp1 and its interactions with other proteins in the context of *C. albicans* biology and pathogenesis.

## Introduction

Although *Candida albicans* is frequently associated with the human host as a commensal, reductions in host immune defense or microbiota can result in the fungus overgrowing its niche and causing disease. The ability of the fungal cell to adhere to host surfaces is a key feature that promotes the opportunity for subsequent disease. Considerable effort has been dedicated to characterization of *C. albicans* cell-surface proteins that are involved in adhesive interactions with host cells, as well as interactions with other microbial cells to form specialized structures such as biofilms (Chaffin, 2008; Ponde et al., 2021).

One notable example of a *C. albicans* cell-surface adhesion protein is Hyphal Wall Protein 1 (Hwp1; Staab and Sundstrom, 1998) that mediates stabilized adhesion between *C. albicans* and human buccal epithelial cells (BECs; Staab et al., 1999). A Gln-Pro-rich region in the N-terminal end of Hwp1 serves as the substrate for host transglutaminases that catalyze the adhesive interaction. Hwp1 is also required for *C. albicans* catheter biofilm formation in a rat model (Nobile et al., 2006) where it is suggested to function in concert with other cell-surface adhesins from the agglutinin-like sequence (Als) family (Nobile et al., 2008).

Development of new tools and reagents advances our understanding of host-pathogen interactions. In that regard, we previously produced MAbs specific for individual proteins in the Als family (Coleman et al., 2009; Coleman et al., 2010; Coleman et al., 2011; Zhao et al., 2011). The theoretically unlimited supply of MAbs provides a considerable advantage over the use of polyclonal antiserum for characterization of protein structure and function. Here, we describe production of an anti-Hwp1 MAb raised against a peptide immunogen from the Hwp1 Gln-Pro-rich adhesive region. We provide a comparative genomics analysis of Hwp1 and related proteins to demonstrate that the Hwp1 Gln-Pro-rich adhesive sequences are only found in *C. albicans* and *Candida dubliniensis*. We also highlight key questions about Hwp1 structure and function that can be addressed using the new anti-Hwp1 MAb.

## Materials and Methods

### Microbial Strains and Culture Conditions

Wild-type *C. albicans* strain SC5314, isolated from the blood of a patient with disseminated disease (Gillum et al., 1984), was purchased from the American Type Culture Collection. Strain CAI12, a *ura3/URA3* derivative of SC5314 (Porta et al., 1999), was a gift from William Fonzi (Georgetown University). Strains CAH7-1A1E2 (*hwp1/hwp1*; Sundstrom et al., 2002) and CAHR3 (*hwp1/hwp1::HWP1*; Staab et al., 1999) were a gift from Paula Sundstrom (Dartmouth University). *Candida dubliniensis* CD36 was a gift from Derek Sullivan (Trinity College). *Candida tropicalis* MYA-3404 and *Candida parapsilosis* CDC 317 were purchased from the American Type Culture Collection. Strains were stored at −80°C in 38% glycerol and routinely cultured on YPD agar (per liter: 10 g yeast extract, 20 g peptone, 20 g glucose, 20 g Bacto agar). Cultures were stored at 4°C for no more than one week before a fresh plate was prepared. YPD liquid medium used the recipe above, excluding the Bacto agar.

### Monoclonal Antibodies

The immunogen for raising a monoclonal anti-Hwp1 was the peptide CDNPPQPDQPDDN (amino acids 154 to 166 of the protein encoded by GenBank sequence U64206). The peptide was synthesized and purified at the Protein Sciences laboratory of the Roy J. Carver Biotechnology Center, University of Illinois at Urbana-Champaign. Methods for inoculation of mice, hybridoma formation, and enzyme-linked immunosorbent assay (ELISA) screening were published previously (Coleman et al., 2009) and summarized here. Mice were immunized intraperitoneally with 0.5 mg of the peptide emulsified in TiterMax for the first injection and 0.25 mg of peptide in incomplete Freund’s adjuvant for the final three injections. Injections were administered at a three-week interval. ELISAs were used to monitor mouse immune response, as well as to screen hybridoma subclones. ELISA plates were coated in free peptide. The anti-Hwp1 MAb was named 2-E8. The Monoclonal Antibody Isotyping Kit (Pierce) was used to determine that MAb 2-E8 was IgG_1_ with a kappa light chain. Anti-Als1 1-B2 (Coleman et al., 2010), anti-Als3 3-A5, and anti-Als6 6-A1 (Coleman et al., 2009) were also used in this study. MAbs were purified by Protein G column chromatography, then concentrated and dialyzed against DPBS using an Amicon filtration device. Protein concentration was measured using the Micro BCA Protein Assay Kit (Thermo Scientific).

### Immunolabeling Methods

Detailed methods for culturing and immunolabeling *C. albicans* cells were described by Coleman et al. (2009). Immunogold electron microscopy methods were also detailed in the same manuscript. A portion of the methods are reproduced here for the reader’s convenience. A single colony of each *C. albicans* strain was inoculated into 10 ml YPD liquid medium in a 50 ml Erlenmeyer flask and incubated for 16 h at 37°C and 200 rpm. Cells were collected by centrifugation, washed twice in sterile water, counted using a hemacytometer and released into pre-warmed RPMI 1640 culture medium with L-glutamine (RPMI medium; Invitrogen catalog number 11875) at a density of 5 x 10^6^ cells/ml. Cultures were incubated at 37°C and 200 rpm shaking for 90 min. Germ tubes were collected by filtration over a 0.22 μm pore-size filter and fixed in 3% paraformaldehyde. Paraformaldehyde fixative was diluted from 16% Electron Microscopy Sciences Paraformaldehyde Aqueous Solution (Fisher Scientific Catalog number 50-980-487) using Gibco Dulbecco’s Phosphate Buffered Saline without calcium or magnesium (DPBS; Thermo Fisher Scientific catalog number 14-190-144).

Fixed *C. albicans* cells were washed 3 times in DPBS prior to and between each step in the immunolabeling protocol. Cells were resuspended in 15 μl/ml normal goat serum for 15 min at room temperature to block non-specific antibody binding. Cells were incubated in 18 μg/ml Protein G-purified MAb in DPBS for 60 min at 4°C on a rotating mixer and then in 3 μg/ml fluorescein isothiocyanate (FITC)-conjugated goat anti-mouse IgG F(ab′)2 fragment-specific antibody (Jackson ImmunoResearch 115-096-006) under the same conditions. Wet mounts of immunolabeled cells were examined using an Olympus BX50 microscope equipped with the Olympus Fluoview FV1200 confocal system and Melles Griot Argon (488 nm) laser. An UP-PLAN 100X Oil objective with a Nomarski prism was used for all images that were captured with Olympus Fluoview software.

A similar method was used to grow cells of different *Candida* species to assess immunolabeling with anti-Hwp1 MAb 2-E8. Growth in RPMI medium did not necessarily induce germ tube formation in other *Candida* species. Hwp1 production in *C. dubliniensis* CD36 was also assessed following growth in conditions demonstrated to increase *HWP1* expression, specifically 10% serum in MilliQ water (Caplice and Moran, 2015). A single colony from a YPD plate of strain CD36 was inoculated into 10 ml of YPD and incubated at 30°C for 18 h with 200 rpm shaking. Cells were washed twice in sterile MilliQ water and counted using a hemacytometer. Washed CD36 cells were resuspended at a density of 1 x 10^7^ cells/ml in 20 ml of prewarmed 10% fetal bovine serum (FBS) in MilliQ water. The culture was incubated for 2 h at 37°C and 200 rpm shaking. Cells were harvested by centrifugation, washed twice in DPBS, and fixed in 3% paraformaldehyde for 10 min. Cells were washed with DPBS and immunolabeling completed as described above. After the final wash step, wet mount cells were visualized using a Keyence All-in-One Fluorescence Microscope (Model BZ-X800E) using the auto-focusing, auto-exposure, and optical sectioning options.

Immunofluorescence assays with multiple antibodies used the Alexa Fluor™ 594 Protein Labeling Kit (Invitrogen) to label purified anti-Als3 3-A5 and the Alexa Fluor™ 633 Protein Labeling Kit (Invitrogen) to label purified anti-Als1 1-B2 according to the manufacturer’s instructions. The CAI12 YPD yeast starter culture was grown as described above. Cells were washed, counted, and released into pre-warmed RPMI medium for 70 min at 37°C and 200 rpm shaking. Cells were fixed in paraformaldehyde, washed, and allowed to adhere to a standard glass microscope slide. Cells were blocked with normal goat serum as described above, then treated with 18 μg/ml of Protein G-purified anti-Hwp1 in DPBS. Each incubation was conducted in a humidified chamber at 4°C for 1 h. The slide was washed three times with DPBS between each immunolabeling step. The slide was first treated with FITC-conjugated goat anti-mouse IgG as described above, then with Alexa Fluor 594-labeled anti-Als3 and Alexa Fluor 633-labeled anti-Als1 at concentrations of 20 μg/ml. ProLong Gold Antifade Mountant (Thermo Fisher Scientific) and a coverslip were added to the slide and cured for 24 h before viewing microscopically on the Olympus FV1200 Confocal System.

### Atomic Force Microscopy Measurements Using Anti-Hwp1

*C. albicans* CAI12 cells were grown in liquid YPD medium overnight at 30°C with 200 rpm shaking. Cells were collected by centrifugation and then resuspended in RPMI 1640 medium at 37°C for 90 min to promote germ tube formation. Germ tubes were washed and resuspended in phosphate-buffered saline (PBS) then deposited by capillarity on polydimethylsiloxane (PDMS) stamps for atomic force microscopy (AFM) observation as described by Dague et al. (2011). AFM tips were coated with dendritips as previously described (Jauvert et al., 2012) to ensure subsequent fixation of the anti-Hwp1 MAb. The AFM JPK Nanowizard III from JPK Instruments (Berlin) was used in Quantitative Imaging mode, with 20 ms approach and retract time, 2 µm z length, and 0.5 nN maximum applied force (Chopinet et al., 2013).

### Bioinformatics Methods

*C. albicans* sequences were downloaded from the *Candida* Genome Database (CGD; http://www.candidagenome.org; Skrzypek et al., 2017). “A” alleles from the diploid genome assembly were used preferentially. Database searches used BLAST *via* the utility on either the CGD or NCBI website (https://blast.ncbi.nlm.nih.gov/Blast.cgi). European Bioinformatics Institute (EMBL-EBI) tools were used for translating nucleotide sequences, sequence alignment, and other general processes (https://www.ebi.ac.uk/services; Cook et al., 2017). SignalP-6.0 Server (http://services.healthtech.dtu.dk/service.php?SignalP; Nielsen, 2017) was used to locate putative secretory signal peptides. The big-PI Fungal Predictor GPI Modification Site Prediction in Fungi was used to locate putative GPI addition sites (https://mendel.imp.ac.at/gpi/fungi_server.html). The *Candida* Gene Order Browser (CGOB; http://cgob.ucd.ie; Maguire et al., 2013) was used to assess ortholog groups based on synteny.

Protein structural predictions derived using AlphaFold (Jumper et al., 2021) were downloaded from the AlphaFold Protein Structure Database (https://www.alphafold.ebi.ac.uk) or derived using AlphaFold Colab (AlphaFold v2.1.0; https://colab.research.google.com/github/deepmind/alphafold/blob/main/notebooks/AlphaFold.ipynb). Structural visualization and alignments used the PyMOL Molecular Graphics System, Version 2.5.2 (Schrödinger, LLC).

### DNA Sequence Correction

Putative orthologs of *C. albicans HWP1*, *RBT1*, and *HWP2* were identified from online databases as described above. The resulting sequences were examined for the presence of expected structural features such as a secretory signal peptide at the N-terminus of the predicted protein and a glycosylphosphatidylinositol (GPI) anchor addition site at the C-terminal end. Lack of such features suggested that the sequences may have errors that altered a predicted start site or caused a premature stop codon. Other sequences had unknown information (e.g. NNN) in the center of the coding region. To derive complete, accurate sequences for these genes, PCR primers were designed using the PrimerQuest Tool (https://www.idtdna.com/pages/tools/primerquest) and synthesized by Integrated DNA Technologies (Coralville, IA). Q5 High Fidelity DNA Polymerase (New England Biolabs) was used according to the manufacturer’s instructions to amplify the problematic regions. PCR products were purified using the MultiScreen HTS 96-well Filtration System (Millipore). Sanger sequencing was completed at the Roy J. Carver Biotechnology Center DNA Services Lab (University of Illinois at Urbana-Champaign, Urbana, IL). Three sequences from *C. orthopsilosis* required correction: *CORT_0E03570* (**Supplementary File S1**), *CORT_0E03560* (**Supplementary File S2**), and *CORT_0E05950* (**Supplementary File S3**). Complete experimental details and accession numbers for the corrected sequences are provided in the **Supplementary Files**.

### Adhesion Assay

The method for assessing antibody blocking of *C. albicans* adhesion to freshly collected human buccal epithelial cells (BECs) was described previously (Beucher et al., 2009) as a modification of a protocol from Zhao et al. (2004) and is reproduced below. Involvement of human subjects in the research project was reviewed and approved by the Office for the Protection of Research Subjects, Biomedical Research Institutional Review Board, University of Illinois at Urbana-Champaign. Informed consent was secured in writing from each study participant. Because of its lack of a signal in immunofluorescence assays (indicating a low concentration of protein on the *C. albicans* cell surface; Coleman et al., 2009; Coleman et al., 2010), the anti-Als6 MAb was used as a negative control for adding protein to the adhesion assays.

Assays were conducted in duplicate on three separate days. A total of 2 × 10^6^ YPD-grown, DPBS-washed CAI12 yeast cells were inoculated into 4 ml RPMI medium in a 25-ml Erlenmeyer flask. Cultures were incubated at 37°C and 200 rpm shaking to induce germ tube formation for 30 min. MAb was added to the flask at a final concentration of 20 µg/ml. Flasks to which only DPBS was added were also included each day. Flasks were incubated for 30 min and then 2 × 10^4^ BEC added to each. Following incubation for 30 min at 37°C and 200 rpm shaking, BECs were collected onto 12 µm pore-size filters (Nucleopore polycarbonate filters; Corning catalog number 111116), washed, transferred to glass microscope slides, heat fixed and stained with crystal violet. The total number of germ tubes adherent to 50 BECs was counted and expressed as the mean number of germ tubes per BEC.

A mixed model analysis of variance was used to assess the differences between treatments. Day, replicate (rep), treatment (trt), and mean number of germ tubes per BEC (gt) were considered in the analysis. Data were analyzed using PROC MIXED in SAS (SAS Institute, Cary, NC). Separation of means was performed with the LSMEANS option. Program commands included: data BEC; input day trt rep gt @@; proc mixed data = BEC; class day trt; model gt = trt; random day day*trt; lsmeans trt/pdiff.

## Results

**Figure 1:**
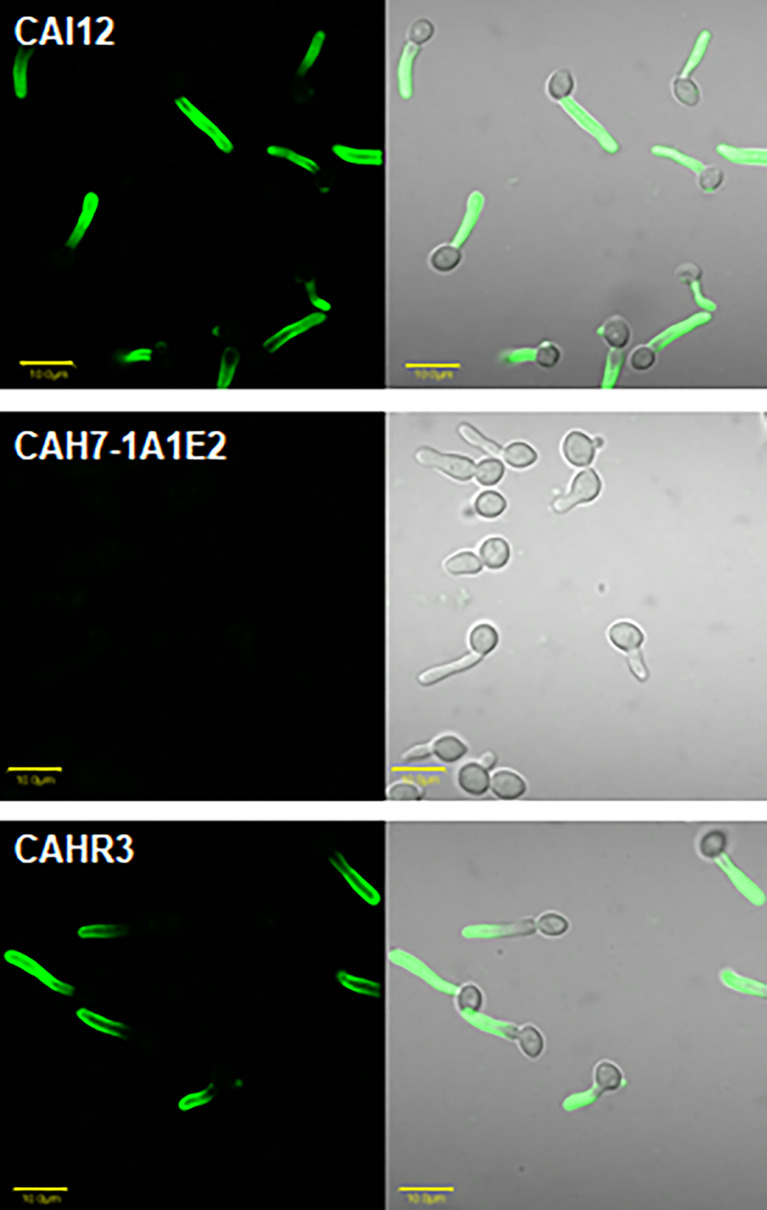
Immunolabeling of *C. albicans* cells to demonstrate specificity of anti-Hwp1 MAb 2-E8. Immunolabeling using the anti-Hwp1 MAb 2-E8 and a FITC-labeled goat anti-mouse secondary antibody only produced a visible signal in *C. albicans* strains that had at least one *HWP1* copy. Immunolabeling was visible only on the germ tube, not the yeast cell, consistent with previously reported *HWP1* expression patterns (Staab et al., 1996). The scale bar in each image denotes 10 μm.

**Figure 2:**
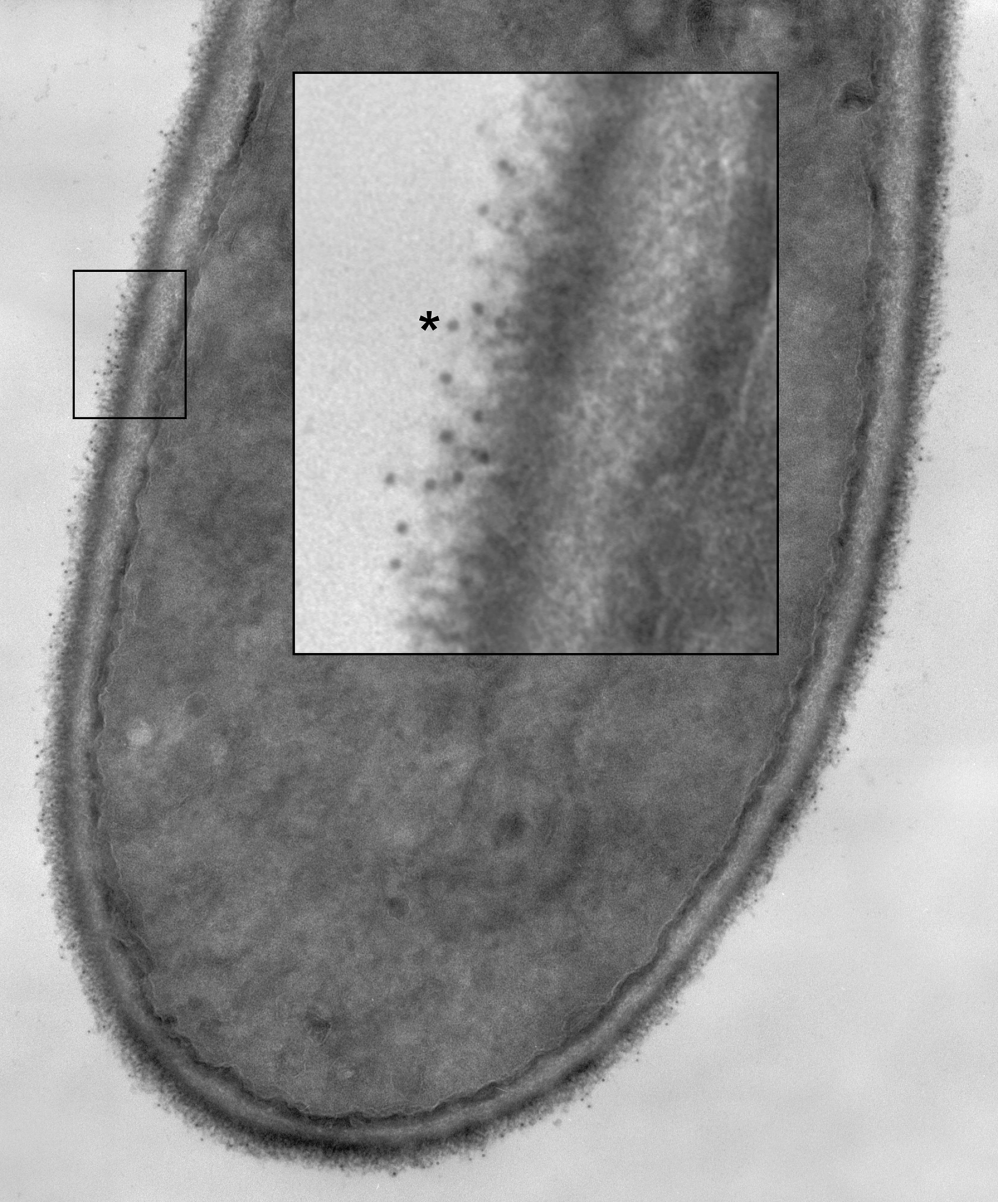
A *C. albicans HWP1/HWP1* germ tube labeled with anti-Hwp1 MAb 2-E8 and a secondary anti-mouse, gold-conjugated antibody. Gold particles, marking the location of Hwp1, appeared in the micrograph as small, electron-dense dots distributed over the outermost surface of the germ tube. A portion of the image was enlarged (inset) to better visualize the gold particles. One gold particle was marked with an asterisk and over a dozen others were visible in the enlarged image.

Specificity of anti-Hwp1 MAb 2-E8 for Hwp1 on the *C. albicans* cell surface was demonstrated by immunolabeling wild-type *C. albicans* cells, a *hwp1/hwp1* mutant (CAH7-1A1E2), and *hwp1/hwp1::HWP1* reintegrant strain (CAHR3; **Figure 1**). MAb 2-E8 labeled the germ tube of strain CAI12 (*HWP1/HWP1*), but not strain CAH7-1A1E2 (*hwp1/hwp1*). Reintegration of a wild-type *HWP1* allele in strain CAHR3 restored MAb 2-E8 immunolabeling, consistent with the conclusion that the MAb is specific for Hwp1 in *C. albicans* cells. Use of anti-Hwp1 MAb 2-E8 for immunogold electron microscopy showed gold particles distributed across the germ tube surface, associated with the flocculant, outermost layer of the cell (**Figure 2**).

**Figure 3:**
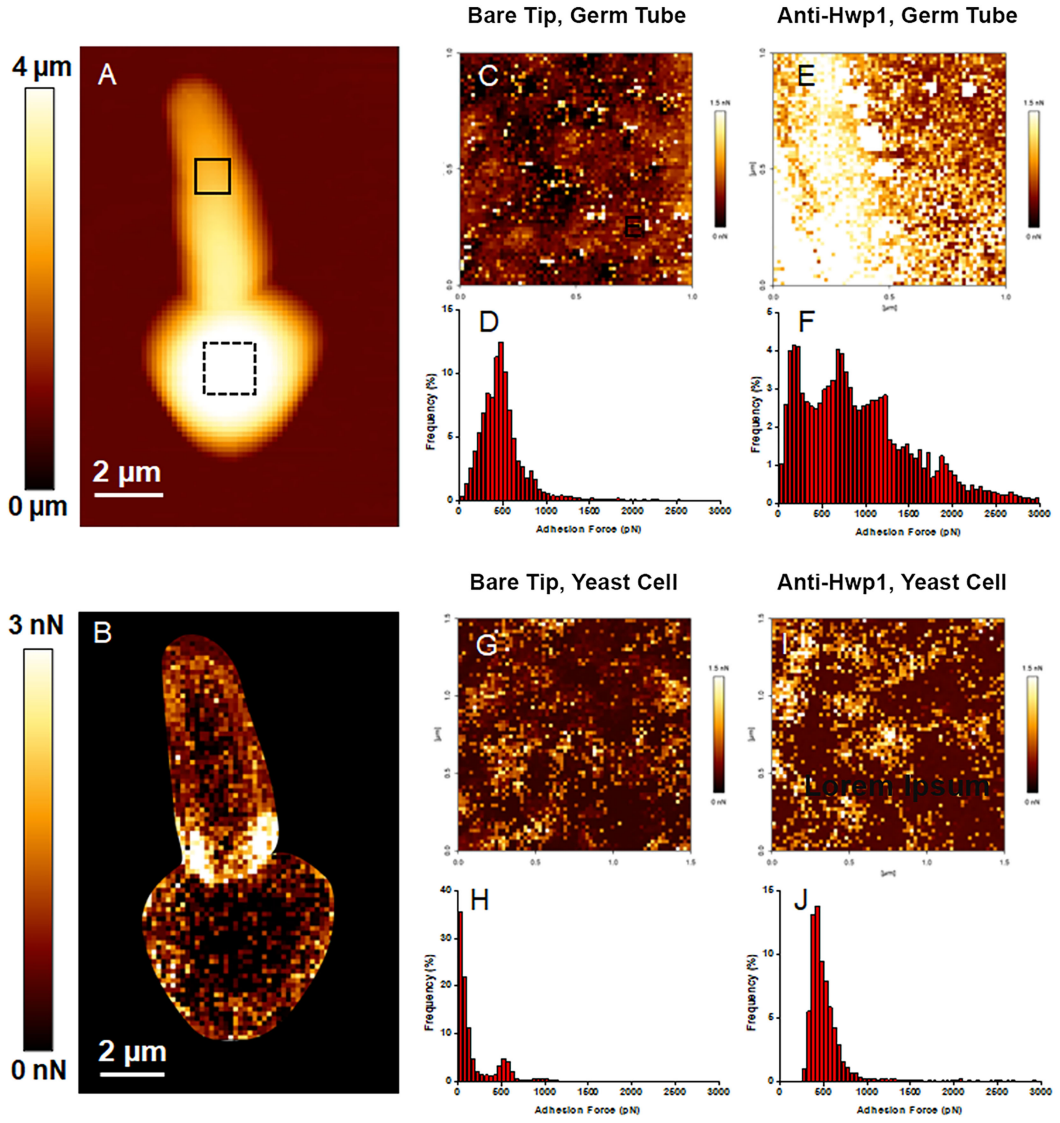
AFM analysis of a *C. albicans* CAI12 germ tube. **(A)** The AFM height image recorded in Quantitative Imaging mode with a color scale to indicate the contact point height (z range), which is the vertical coordinate where the AFM tip touches the cell surface. The image shows the relief of the yeast and germ tube surfaces with lighter color indicating higher points and darker color indicating lower points. **(B)** The adhesion map of the same cell recorded with a bare AFM tip with a color scale to indicate the adhesion force range. Lighter color indicated a higher occurrence of non-specific adhesive events. These regions were avoided in subsequent experiments to record the adhesion maps. **(C)** The adhesion map, recorded with a bare AFM tip, of the 1 μm x 1 μm solid square on the germ tube surface in **(A)**. **(D)** Repartition of the maximum adhesion measured on the 4096 force curves corresponding to the map in **(C)**. **(E)** The adhesion map, recorded with the anti-Hwp1 functionalized AFM tip, of the 1 μm x 1 μm solid square on the germ tube surface in **(A)**. **(F)** Repartition of the maximum adhesion measured on the 4096 force curves corresponding to the map in **(E)**. **(G)** The adhesion map, recorded with a bare AFM tip, of the 1.5 μm x 1.5 μm dashed square on the yeast cell surface in **(A)**. **(H)** Repartition of the maximum adhesion measured on the 4096 force curves corresponding to the map in **(G)**. **(I)** The adhesion map, recorded with the anti-Hwp1 functionalized AFM tip, of the 1.5 μm x 1.5 μm dashed square on the yeast cell surface in **(A)**. **(J)** Repartition of the maximum adhesion measured on the 4096 force curves corresponding to the map in **(I)**.

MAb 2-E8 was also used for atomic force microscopy (AFM) detection of *C. albicans* cell-surface Hwp1 (**Figure 3**). Single Molecule Force Spectroscopy experiments, conducted with functionalized AFM tips, measured specific interaction forces between one molecule on the functionalized tip and one molecule on the sample surface. Specific molecular interactions were recorded during retraction of the AFM tip from the sample. The surface of the germ tube was scanned with either a bare AFM tip composed of silicon nitride (Si_3_N_4_) (**Figure 3C**) or functionalized tip coated with purified MAb 2-E8 (**Figure 3E**). The same technique was applied to the yeast cell (bare AFM tip in **Figure 3G** and anti-Hwp1 functionalized tip in **Figure 3I**). The adhesion force was determined by measuring the piezo-retraction required to break the interaction between the MAb attached to the tip and the cell surface. This process was repeated while moving the cantilever between each measurement, thus creating a map of the interactions that revealed how Hwp1 was distributed on the cell surface. Histograms representing the repartition of recorded interaction forces are shown in **Figures 3D, F, H, J**, respectively. The intensity and frequency of adhesive events recorded with a bare AFM tip on the surface of the germ tube or yeast cell were very low (**Figures 3D, H**). In contrast, specificity of the antibody-antigen interaction was visible from the remarkably high frequency and intensity signals observed from scanning germ tubes with the anti-Hwp1 functionalized tip compared to those recorded on the yeast cell (**Figures 3F vs. 3J**). Low signals on the yeast cell were consistent with fluorescence microscopy images that suggested abundant representation of Hwp1 only on the germ tube (**Figure 1**).

Genome database searching was used to predict the potential for the anti-Hwp1 MAb 2-E8 to recognize the surface of other fungal species that are closely related to *C. albicans*. *C. albicans HWP1* (C4_03570W_A) is located on Chromosome 4 near two other genes with which it shares sequence similarity: *RBT1* (C4_03520C_A) and *HWP2* (C4_03510C_A). The *Candida* Gene Order Browser (CGOB; Maguire et al., 2013) identified orthologs of all three loci, based on synteny; these results are displayed as vertical alignments of genes with conserved chromosomal location, called pillars. **Table 1** shows the pillars corresponding to *HWP1*, *RBT1*, and *HWP2*. BLAST search results with an E value less than 1.0 were retained in the list. The list also includes loci beyond those identified in the CGOB; those sequences are shown in the far-right column of **Table 1**. Amino acid sequences translated from the **Table 1** loci are included in **Supplementary Table S1** to facilitate examination and construction of sequence comparisons by the reader.

**Table 1:** *HWP1*, *RBT1*, and *HWP2* orthologs in *Candida albicans* and closely related species as displayed in the *Candida* Gene Order Browser (CGOB) and detected using BLAST.

Species	*HWP1* Pillar*	*RBT1* Pillar*	*HWP2* Pillar*	Additional BLAST Hits*
*Candida albicans*	C4_03570W_A	C4_03520C_A	C4_03510C_A	
*Candida dubliniensis*	Cd36_43360	Cd36_43400	Cd36_43420	
*Candida tropicalis*	CTRG_00478	CTRG_00477		
*Candida parapsilosis*	CPAR2_403520	CPAR2_403510		CPAR2_602610
*Candida orthopsilosis*	CORT_0E03570	CORT_0E03560		CORT_0E05950
*Candida metapsilosis*	Cm_5_551924^†^	Cm_5_537075^†^		Cm_4_904913^†^
*Lodderomyces elongisporus*		LELG_04495		
*Meyerozyma guilliermondii*		PGUG_02521		
*Debaryomyces hansenii*		DEHA2G17820g		

* Gene names were assigned to indicate relative location. For example, all three C. albicans genes were on chromosome 4 (C4) with RBT1 (location 3520) adjacent to HWP2 (location 3510), near HWP1 (location 3570). HWP1 was transcribed in the forward direction (W) with RBT1 and HWP1 transcribed in the opposite direction (C). “A” alleles from the Candida Genome Database were used preferentially in the analysis. Gene names for other species similarly denote relative location and indicated that HWP1 and RBT1 were adjacent or near each other in species where both genes were found. The additional BLAST hits were not part of this gene cluster in any species.

^†^The C. metapsilosis genome sequence used in CGOB was not identified in available resources. CGOB used “CMET” designations for gene names. C. metapsilosis BLAST analysis presented here used genome assembly GCA_008904905.1, strain ATCC 96143 (Oh et al., 2019). Gene designations shown above indicated the contig number (i.e. either contig 5 or 4 for the sequences included here) and the starting nucleotide number in the contig sequence. **Supplementary Table S1** shows gene names decoded into the CMET format for entries with available information.

The peptide immunogen used to raise anti-Hwp1 MAb 2-E8 was in the N-terminal region of repeated Gln/Pro-rich sequences that serve as a substrate for mammalian transglutaminases and facilitate stabilized adhesion of *C. albicans* to BECs (Staab and Sundstrom, 1998; Staab et al., 1999). Sequence comparisons and alignments among the Hwp1/Rbt1/Hwp2 orthologs (**Supplementary Table S1**) indicated that the Gln/Pro-rich sequences were unique to Hwp1 in *C. albicans* and *C. dubliniensis* although the *C. dubliniensis* Cd36_43360 sequence that best matched the peptide immunogen was imperfect (**Table 2**). The 13-mer immunogen peptide sequence was used as the query for a tblastn search of the genomes for species listed in **Table 1**. Additional partial sequence matches were revealed (**Table 2**) although the resulting proteins were unlikely to be present on the fungal cell surface.

**Table 2:** Best tblastn matches to the anti-Hwp1 MAb 2-E8 immunogen peptide among species closely related to *C. albicans*.

Species	Locus	Function	Peptide Sequence*
*C. albicans*	*HWP1* C4_03570W_A	Cell adhesion	**CDNPPQPDQPDDN**
*C. dubliniensis*	*HWP1* Cd36_43360	Cell adhesion	**CDNPPQP**EE**P**C**DN**
*C. albicans*	C2_07440C_A	Sterol esterase	E**NPPQ**Q**DQ**
*C. dubliniensis*	Cd36_54350	mRNA binding protein Ssd1	**PP**T**PDQ**SN**DN**
*M. guilliermondii*	PGUG_05369	Phosphatidylinositol 3,5-bisphosphate-binding protein	**NP**S**Q**QE**QPDD**
*M. guilliermondii*	PGUG_03203	Ribitol kinase	**NPPQPD**
*D. hansenii*	DEHA2A06886g	Aromatic amino acid aminotransferase	**NPPQP**EL**P**EN

*The immunogen peptide was derived from the C. albicans Hwp1 sequence and is shown in bold red type. Best-match sequences from other proteins, identified by BLAST, were aligned vertically with the immunogen sequence. Red bold type marked amino acids identical to the immunogen peptide.

**Figure 4:**
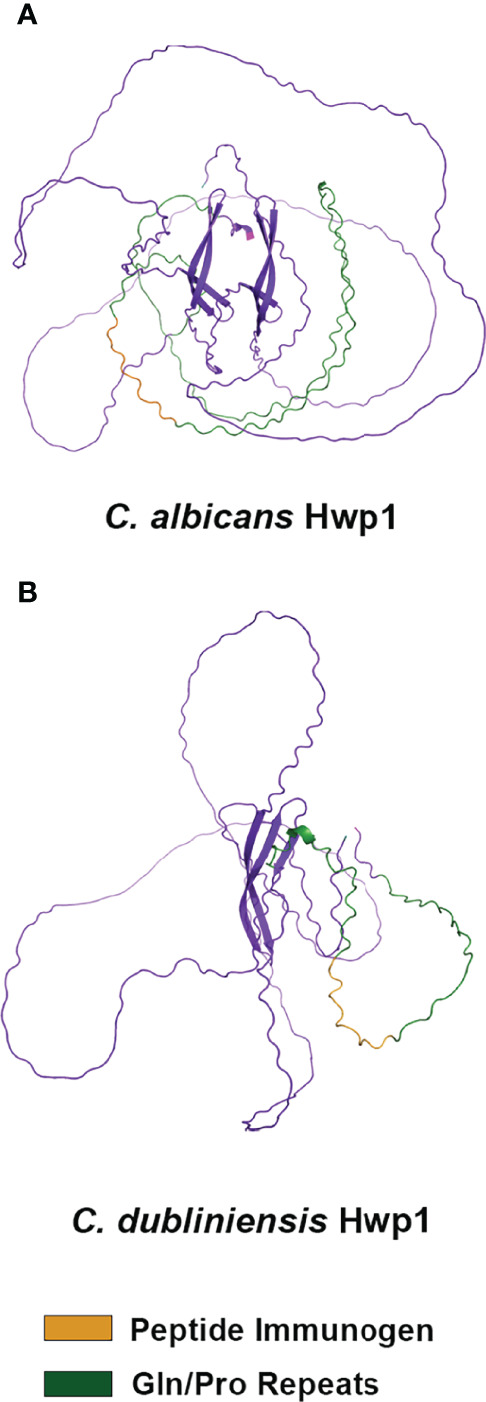
Alpha Fold structural prediction for *C. albicans* Hwp1 and its *C. dubliniensis* ortholog. The *C. albicans* Hwp1 structural prediction **(A)** was downloaded from the Alpha Fold Protein Database [P46593 (HWP1_CANAL)]; AlphaFold Colab was used to generate the structural prediction for *C. dubliniensis* Hwp1 (GenBank XP_002419994; **(B)**. Amino acid sequences were trimmed to reflect cleavage of the secretory signal peptide, as well as processing of the Kex2 and GPI anchor addition sites. AlphaFold predicted a largely unstructured molecule for each but had low confidence in the predictions except for the region of antiparallel beta-sheets in the C-terminal half of the protein. The Gln-Pro-rich repeated sequences (green color) were more extensive in *C. albicans* Hwp1 than in the *C. dubliniensis* protein. Orange color denoted the immunogen used to raise anti-Hwp1 MAb 2-E8, for which 10 of 13 amino acids were conserved in the *C. dubliniensis* protein (**Table 2**).

*C. albicans* Hwp1 was downloaded from the AlphaFold database of predicted protein structures (https://www.alphafold.ebi.ac.uk) to visualize the position of the 13-mer immunogen; AlphaFold Colab was used to predict a structure from the *C. dubliniensis* Hwp1 sequence (**Figure 4**). AlphaFold predictions showed largely unstructured molecules although the predictions did not have a high level of confidence. However, both structures suggested surface-exposure of the immunogen.

**Figure 5:**
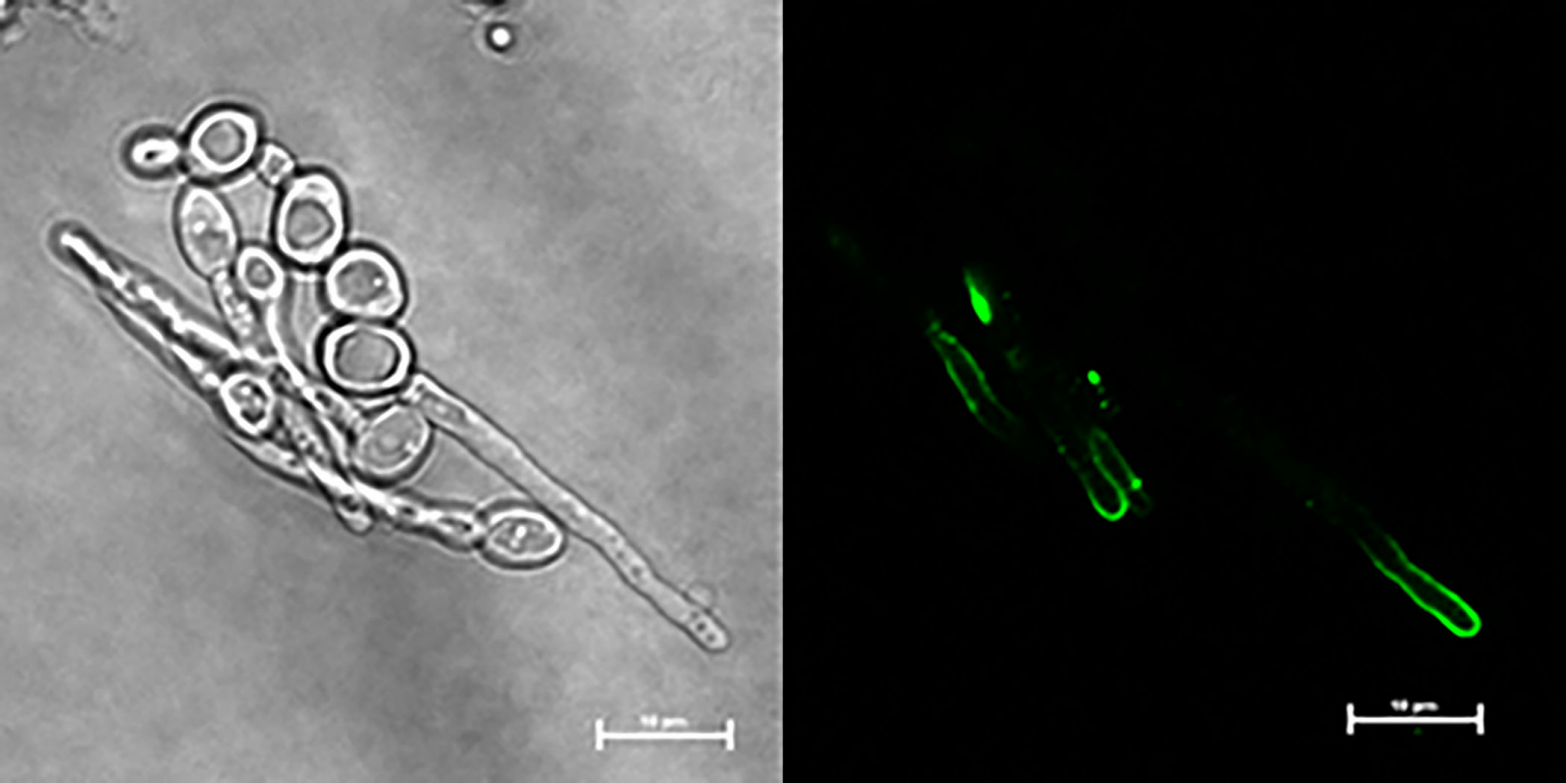
Anti-Hwp1 immunolabeling of *C. dubliniensis* cells. Strain CD36 was grown for 2 h in 10% FBS diluted with MilliQ water, then immunolabeled with anti-Hwp1 MAb 2-E8 and a FITC-conjugated anti-mouse secondary antibody. Signal was observed toward the tip of some germ tubes. The scale bar in each image denotes 10 μm.

*C. dubliniensis* CD36, *C. tropicalis* MYA-3404, and *C. parapsilosis* CDC 317 were tested using the same growth conditions and immunolabeling methods that were successful for *C. albicans*; no anti-Hwp1 signals were observed (data not shown). Negative results for *C. tropicalis* and *C. parapsilsosis* were consistent with genomic information that suggested lack of an epitope that could be recognized by anti-Hwp1 MAb 2-E8 (**Tables 1**, **2** **;** **Supplementary Table S1**). The potential for a Hwp1 epitope to exist in *C. dubliniensis* (**Table 2**) prompted literature searches for alternative growth conditions that might produce sufficient Hwp1 protein for immunolabeling detection. Caplice and Moran (2015) demonstrated a difference in *EFG1* regulation of genes between *C. albicans* and *C. dubliniensis* with *C. dubliniensis* genes showing a lag in serum-induced expression and a requirement for nutrient depletion for maximum expression. *C. dubliniensis* CD36 grown in 10% serum produced visible anti-Hwp1 immunolabeling signal on some germ tubes (**Figure 5**). Immunolabeling was observed primarily toward the germ-tube tip in the longer germ tubes shown.

**Figure 6:**
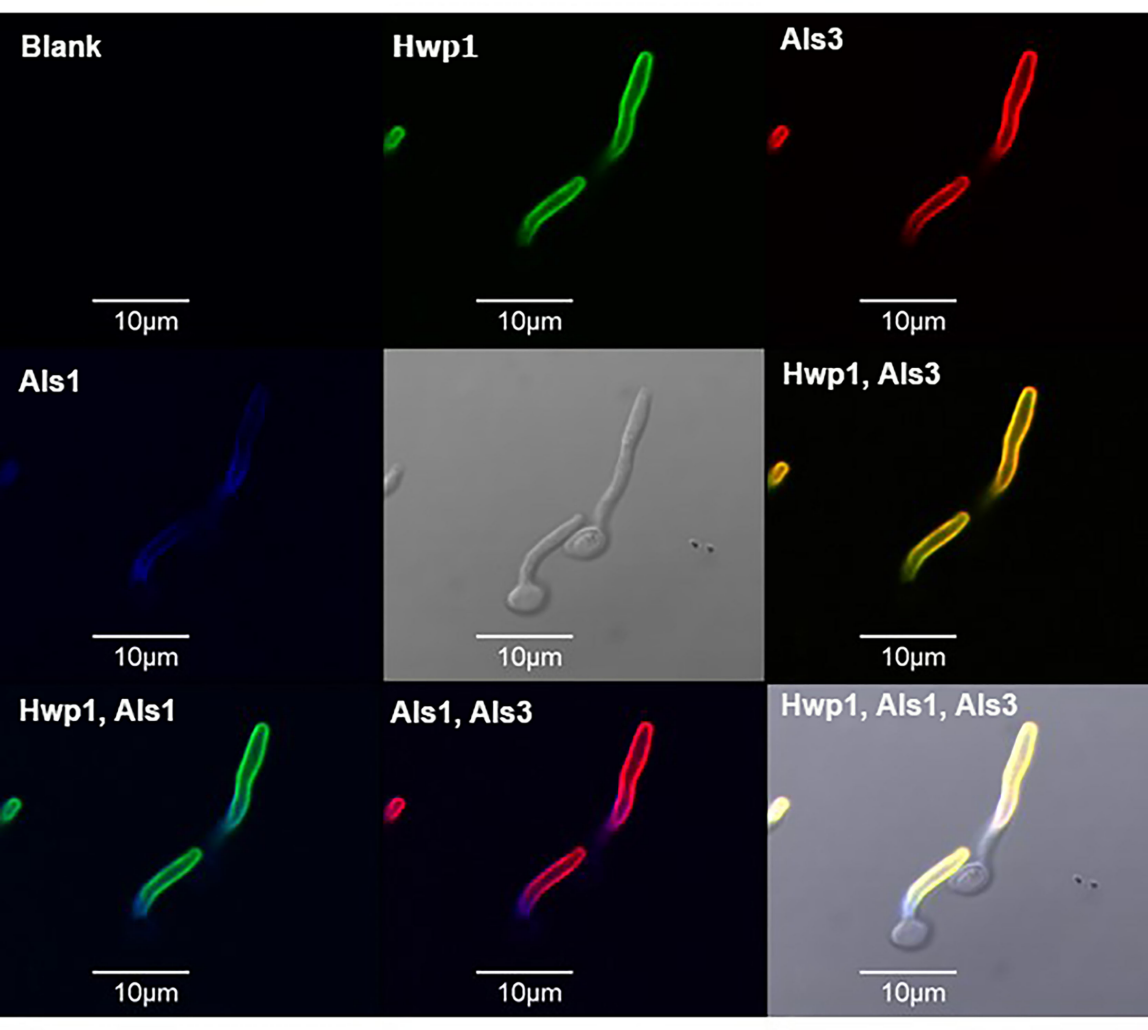
Immunolabeling of *C. albicans* germ tubes with MAbs specific for Hwp1, Als1, and Als3. *C. albicans* strain CAI12 was pre-grown as yeast cells in YPD medium, then washed and transferred to RPMI medium for 70 min to form germ tubes. Germ tubes were labeled with anti-Hwp1 2-E8 followed by a secondary FITC antibody (depicted as a green color). Germ tubes were also directly labeled with Alexa Fluor 594-conjugated anti-Als3 (red color) and Alexa Fluor 633-conjugated anti-Als1 (false-colored blue). Pairs of signals were overlaid in other frames and marked accordingly. The yellow color in the “Hwp1, Als3” panel was the combination of individual signals from each protein, indicating shared localization. In contrast, overlaying either Hwp1 or Als3 with Als1 maintained the original colors for each protein, suggesting less overlap between Als1 with either Hwp1 or Als3. The scale bar in each image denotes 10 μm.

**Figure 7:**
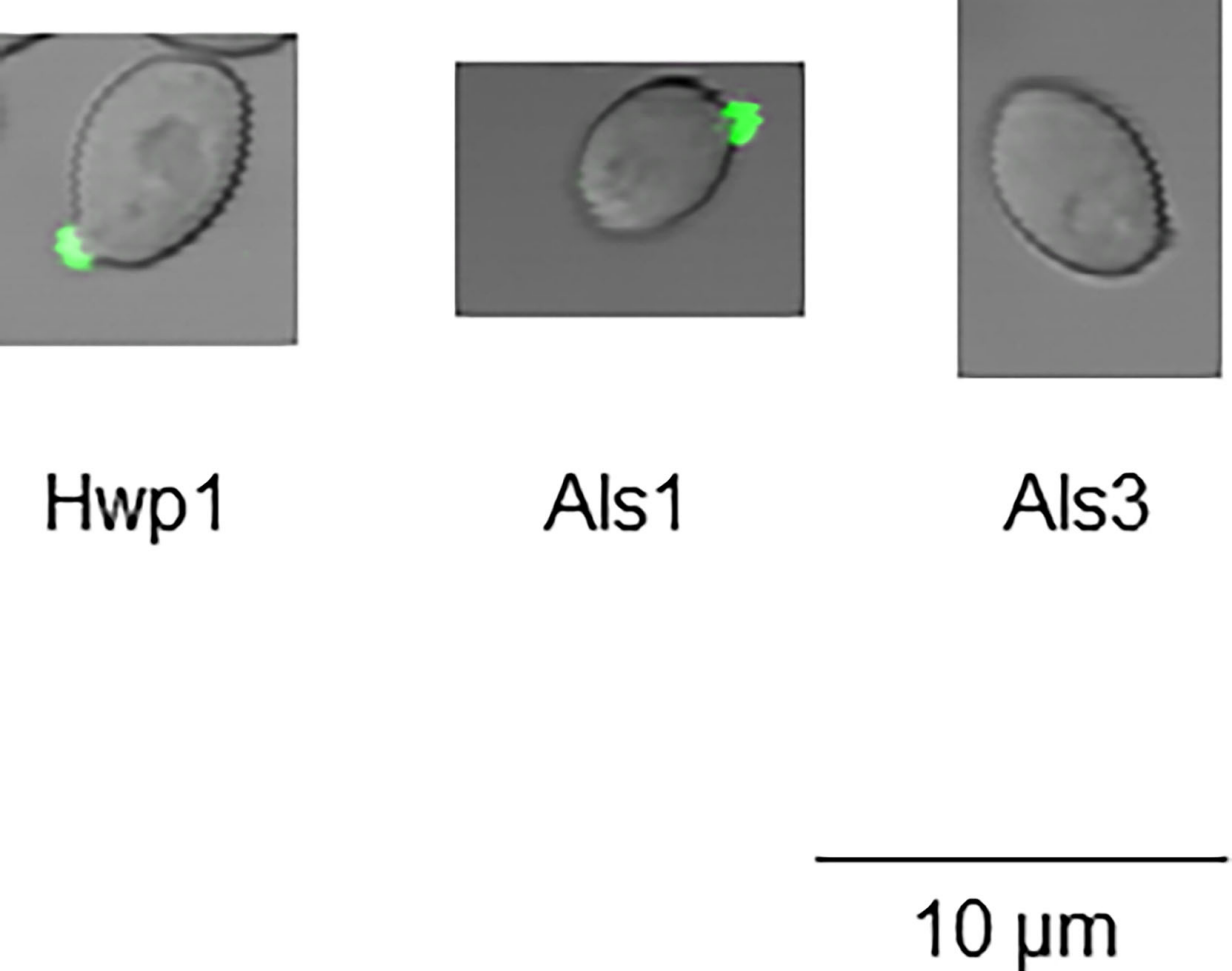
Immunolabeling of *C. albicans* cells 10 min after placing them into RPMI 1640 medium to induce germ tube formation. *C. albicans* CAI12 was grown in YPD to saturation, then washed and placed into RPMI medium at 37°C and 200 rpm shaking. Cells were collected by filtration at various time points with a 10-min time point pictured. Immunolabeling with either anti-Hwp1 (left), anti-Als1 (center), or anti-Als3 (right) was used to follow the time course of protein detection on the surface of yeast cells at the earliest stages of germ-tube formation. At the 10-min time point, Hwp1 and Als1 were visible by immunolabeling while Als3 required additional incubation to produce a detectable signal.

The localization of Hwp1 on *C. dubliniensis* germ tubes varied from *C. albicans* Hwp1 which was distributed over most of the germ tube length (**Figure 6**). *C. albicans* Als1 and Als3 are also known for abundant presence on the germ tube. Previous work showed that *ALS1* expression was detectable before *ALS3* when *C. albicans* yeast cells were placed into RPMI medium to promote germ tube formation (Zhao et al., 2004). That result was replicated here using MAbs specific for each protein (**Figure 7**). Immunolabeling with anti-Hwp1 MAb 2-E8 demonstrated that the kinetics of Hwp1 production were more similar to Als1 than Als3 (**Figure 7**). Although there are regions of the germ tube where all three proteins were present, immunolabeling of longer germ tubes (**Figure 6**), showed obvious Als1 blue false coloring closest to the junction between the mother yeast and germ tube. Therefore, despite the appearance of Als1 before Als3, there is greater overlap between Hwp1 and Als3 on the lengthening germ tube than overlap of either protein with Als1.

**Figure 8:**
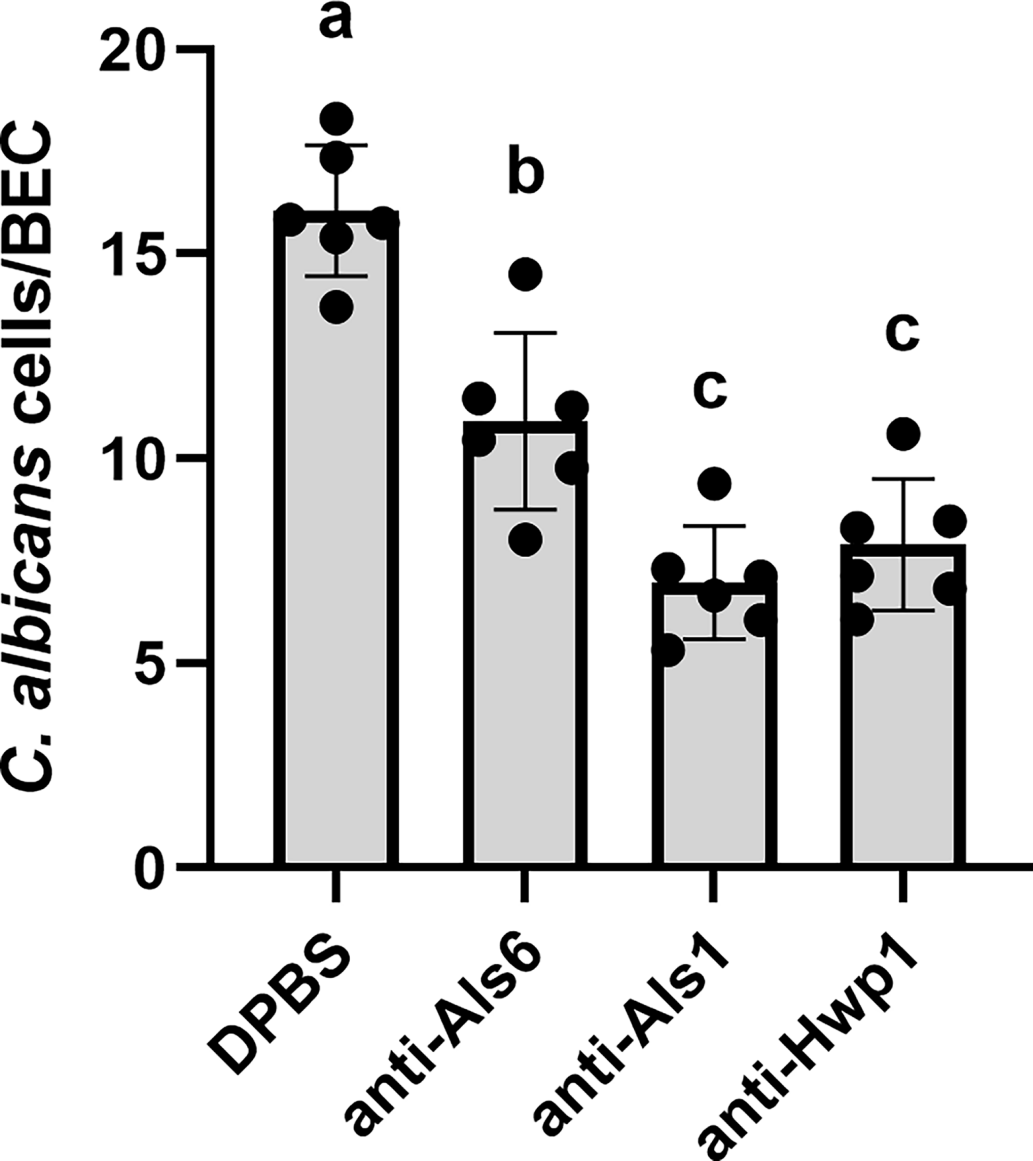
Assessing the ability of anti-Hwp1 MAb 2-E8 to block *C. albicans* adhesion to freshly collected human BECs. DPBS, negative-control protein (anti-Als6), anti-Als1, or anti-Hwp1 were added to flasks containing *C. albicans* CAI12 germ tubes and freshly collected human BECs. Following incubation for 30 min, cells were collected by filtration and transferred to glass microscope slides. The total number of germ tubes adherent to the first 50 BECs visualized was counted. Data displayed in the histogram are mean ± standard deviation from duplicate assays conducted on three independent days. The effect of treatment was significant in the analysis (P = 0.0003). Lowercase letters indicate statistical significance groups. The DPBS treatment was significantly different from anti-Als6 (P = 0.0015), from anti-Als1 (P < 0.0001), and from anti-Hwp1 (P = 0.0001). The anti-Als6 treatment was significantly different from anti-Als1 (P = 0.0054) and anti-Hwp1 (P = 0.0176). The anti-Als1 and anti-Hwp1 treatments were not significantly different from each other (P = 0.3573). Addition of protein (anti-Als6) was sufficient to decrease adhesion compared to DPBS alone. Addition of anti-Als1 or anti-Hwp1 further decreased adhesion indicating that each MAb blocked the adhesive interaction between *C. albicans* germ tubes and human BECs.

The Gln-Pro-rich region against which the anti-Hwp1 MAb 2-E8 was raised mediates stabilized adhesion of *C. albicans* to human BECs (Staab and Sundstrom, 1998; Staab et al., 1999). Assays were conducted to test whether MAb 2-E8 could block the adhesive interaction. Anti-Als1 MAb 1-B2 was demonstrated previously to block adhesion of *C. albicans* to BECs and was included as an assay control (Coleman et al., 2010). Anti-Als6 MAb 6-A1, which does not have detectable anti-adhesive activity, was included as a negative control for the effect of adding protein to the assay (Coleman et al., 2009). Anti-Hwp1 MAb 2-E8 at a concentration of 20 µg/ml was sufficient to block adhesion, similar to anti-Als1. (**Figure 8**).

## Discussion

Immunization of mice with a peptide derived from the Gln-Pro-rich adhesive domain of *C. albicans* Hwp1 was used to create MAb 2-E8, which is specific for Hwp1 in *C. albicans* (**Figures 1**–**3**). MAb 2-E8 also recognizes the surface of *C. dubliniensis* germ tubes (**Figure 5**), suggesting cross-reactivity with a similar sequence in *C. dubliniensis* Hwp1 (**Table 2**). Previous literature reports described a polyclonal rabbit antiserum raised against an Hwp1 fragment heterologously expressed in *Pichia pastoris* (Staab et al., 1996). The antiserum was used to demonstrate Hwp1 features such as its selective detection on *C. albicans* germ tubes (Staab et al., 1996), a result confirmed here using MAb 2-E8 for immunolabeling and AFM analysis (**Figures 1**, **3**).

AFM analysis demonstrated highly specific mapping of a native cell wall protein on the *C. albicans* surface using tips functionalized with a MAb. Such measurements are more specific than use of tagged proteins (Formosa et al., 2015) or polyclonal antibodies (Beaussart et al., 2012) that are likely to cross react with other surface proteins. Non-specific events that were recorded in experiments reported here are mediated by other *C. albicans* surface proteins that display different profiles from the specific antigen-antibody interactions (**Figure 3**). For example, the highly adhesive *C. albicans* surface that creates non-specific interactions with a bare AFM tip were observed for Pga proteins (Cabral et al., 2014). Another notable outcome of this work is that Hwp1 is observed earlier during germ tube formation using immunofluorescence than by AFM. Hwp1 was observed on the *C. albicans* surface using immunofluorescence (**Figure 7**) but required longer germ tubes (approximately 90 to 120 min in inducing medium) for AFM observations. This difference may be due to MAb ability to access Hwp1 in solution for immunofluorescence compared to attachment to the dendrimer linker structure on the AFM tip, which is a very large structure compared to the cell wall network.

Examination of Hwp1 ortholog sequences among species closely related to *C. albicans* suggested that the Gln-Pro-rich repeated region that is responsible for stabilized adhesion between *C. albicans* and human BECs is only present in *C. albicans* and *C. dubliniensis* (**Supplementary Table S1**). In *C. albicans*, *HWP1* is located on chromosome 4 near other similar genes named *RBT1* and *HWP2*. Similarity among *C. albicans* Hw1p, Rbt1, and Hwp2 is found in the signal peptide and C-terminal region. Sequence alignments suggest that the Gln-Pro region is essentially a little adhesive cassette dropped into *C. albicans* Hwp1 and preserved in *C. dubliniensis* Hwp1. Orthologs of Hwp1 in other species, as well as Rbt1, Hwp2 and their orthologs do not have that cassette. The presence of Hwp1 clearly contributes to the greater adhesive capacity of *C. albicans* compared to other closely related species (Calderone and Braun, 1991). Indeed, blocking the adhesive domain with MAb 2-E8 resulted in a significant reduction in *C. albicans* adhesion to BECs (**Figure 8**).

Despite the close relationship of the species *C. albicans* and *C. dubliniensis*, differences in transcriptional regulation of germ tube/hypha growth are documented between them. For example, the regulation of *HWP1* transcription by Efg1 differs between the species (Caplice and Moran, 2015). In *C. dubliniensis*, *HWP1* expression lagged behind the kinetics of *HWP1* expression in *C. albicans*. Approximately 2 h of growth induction in nutrient-limited conditions were required for maximum *HWP1* expression levels to be reached. Anti-Hwp1 MAb 2-E8 detected protein at the tips of growing hyphae (**Figure 5**), rather than along the full length of the germ tube (**Figure 6**). Additionally, not all *C. dubliniensis* germ tubes produced a positive signal with anti-Hwp1 MAb 2-E8, another difference between the species. Availability of MAb 2-E8 can be leveraged to better understand how germ tube formation varies between the species and how differential protein abundance and localization may affect pathogenesis.

The Hwp1 Gln-Pro-rich sequences are present in multiple repeated copies (Staab et al., 1999), presumably each with potential to participate in adhesive interactions with BECs. Other *C. albicans* repeat-containing adhesins can have often-extreme allelic variation (Zhang et al., 2003; Hoyer et al., 2008; Oh et al., 2021), leading to questions about diversity of *HWP1* alleles across *C. albicans* isolates. Analysis of *HWP1* restriction fragment length polymorphisms was proposed as a diagnostic assay to distinguish *C. albicans* from *C. dubliniensis*, suggesting stability of fragment size, and therefore repeat copy number across isolates in the same species. Romeo et al. (2006) described PCR primers that amplified the 5’ end of *HWP1* (encompassing the entire Gln-Pro-repeat-containing region) to distinguish between *C. albicans* and *C. dubliniensis* and tested them on approximately 20 isolates of each species. Size of the amplified fragments was consistent within each species with *C. dubliniensis HWP1* having a considerably shorter Gln-Pro-repeat-encoding region than *C. albicans HWP1*. Padovan et al. (2009) described a novel *C. albicans HWP1* allele (called *HWP1-2*) that lacked copies of sequences encoding the Gln-Pro repeat as well as a portion of the Ser-Thr-rich C-terminal region of the protein. A *C. albicans* strain that had two *HWP1-2* alleles was defective in biofilm formation. Although variant *HWP1* alleles have been described, selection seems predominantly for a consistent *HWP1* allele size both in *C. albicans* and *C. dubliniensis*. Hwp1 adhesive function would require sufficient Gln-Pro copies, as well as an overall protein length to display the region on the cell surface. It seems highly likely that anti-Hwp1 MAb 2-E8 should recognize all *C. albicans* and *C. dubliniensis* isolates. Immunofluorescence, immunogold electron microscopy, and AFM techniques used here support the idea that the Gln-Pro region is displayed prominently on the *C. albicans* cell surface (**Figures 1**–**3**).

The AlphaFold algorithm uses deep multiple sequence alignments and information from Protein Database structure templates to arrive at structural predictions that have unprecedented accuracy (Jumper et al., 2021). The AlphaFold database (www.alphafold.ebi.ac.uk) includes the entire *C. albicans* proteome; the user-friendly AlphaFold Google Colab (see **Materials and Methods**) can predict structures from proteins yet to appear in the AlphaFold database. While AlphaFold is viewed as game-changing in its ability to shed light on structural questions, the algorithm did not arrive at the same conclusions for Hwp1 as previous biochemical analysis. AlphaFold predicted Hwp1 to be largely unstructured (**Figure 4**; https://www.alphafold.ebi.ac.uk/entry/P46593) but assigned a low or very low per-residue confidence score to the prediction. Staab et al. (2004) used traditional biochemical analysis of Hwp1 to infer structural properties. Circular dichroism spectra and titration of thiols led to prediction of a disulfide-cross-linked coiled-coil structure for the N-terminal adhesive portion of the protein (amino acids 40-187). In contrast, AlphaFold did not predict disulfide bonds among the 11 Cys residues in this portion of the Hwp1 sequence. Additional biochemical characterization by Staab et al. (2004) demonstrated Hwp1 modification by N- and O-glycosylation, as well as transient addition of a GPI anchor that directed Hwp1 linkage to β-glucan in the *C. albicans* cell wall (Lu et al., 1994).

In addition to its function in stabilized adhesion of *C. albicans* to human BECs, Hwp1 plays other key roles that support colonization and pathogenic potential in *C. albicans*. For example, Hwp1 is required for *in vivo* catheter biofilm formation using a rat model (Nobile et al., 2006). Subsequent investigation led to the hypothesis that Hwp1 promotes *C. albicans* biofilm formation through interaction with Als1 and/or Als3 (Nobile et al., 2008). Although Als1 and Hwp1 can be detected first on the *C. albicans* surface at the site of germ tube emergence *in vitro* (**Figure 7**), the eventual protein distribution on the germ tube shows Als1 closest to the mother yeast and fading as the germ tube elongates (**Figure 6**). Immunolabeling of hyphae isolated from a mouse oral candidiasis model indicated that the *in vivo* pattern of Als1 distribution is more homogeneous over the germ-tube length, likely from differential regulation of transcription in the animal (Coleman et al., 2011). It is reasonable to assume a similar Als1 distribution would occur in the rat catheter biofilm model. In cultured cells, Hwp1 is present strongly along the *C. albicans* germ tube length, similar to Als3 (**Figure 6**). *In vitro* or *in vivo*, there is a sizable length of the germ tube on which Hwp1, Als1, and Als3 are co-localized in abundance with obvious opportunity to contact each other.

The literature contains speculation about the nature of the contact between Hwp1 and the Als proteins, suggesting that the interaction may have evolved from that between alpha- and **a**-agglutinin, the mating agglutinins in *S. cerevisiae* (Nobile et al., 2008; Soll, 2008). In *S. cerevisiae*, the monomeric, cell-wall-localized alpha-agglutinin binds with high affinity to the flexible C-terminal end of the small subunit (Aga2; 69 amino acids total in the mature protein) of the heterodimeric **a**-agglutinin (Dranginis et al., 2007). Aga2 is disulfide bonded to the Aga1 anchor protein that displays Aga2 on the cell surface. The idea that Als proteins are orthologs of *S. cerevisiae* alpha-agglutinin has gained support as the number of genomic sequences continues to grow (Oh et al., 2021). For example, CGOB predicts that *S. cerevisiae SAG1* is syntenic with a *C. dubliniensis ALS* locus. Also, Sag1 and Als proteins appear to share a similar structure of the N-terminal domain that is responsible for adhesive (ligand-binding) function (Oh et al., 2021). In contrast, the idea that Hwp1 is an ortholog of *S. cerevisiae* **a**-agglutinin (Aga1) seems prompted by the observation that Hwp1 production and localization during *C. albicans* mating is specific to the **a/a** portion of the conjugation bridge (Daniels et al., 2003). **Table 1** demonstrates that the *HWP1* locus has a rather evolutionarily limited synteny pillar and database searching suggests that there does not appear to be a homolog of *S. cerevisiae AGA1* in *C. albicans*. Alternatively, the nature of the interaction between Hwp1 and Als proteins may not involve ligand-binding but other mechanisms such as aggregation. Hwp1 contains sequences with amyloid-forming potential that could catalyze aggregative interactions with other abundant cell-surface proteins (Ramsook et al., 2010). It remains to be demonstrated whether Hwp1 glycosylation (Staab et al., 2004) obscures potential amyloid-forming sequences or if they are available to access similar sequences in other proteins and contribute to aggregative interactions.

Several tools and reagents have been developed recently that make it possible to test the hypotheses presented above. For example, the nature of the Als peptide-binding activity is characterized at the molecular level (Salgado et al., 2011). *C. albicans* strains that display functionally mutant versions of Als3 on the surface under control of the native promoter were described (Lin et al., 2014). One strain incorporates mutations in the peptide-binding cavity that abolish ligand-binding function while still displaying the structurally correct full-length, cell-surface Als3 under control of its native promoter. Another strain displays an Als3 molecule in which the amyloid-forming (aggregative) function is disrupted, but ligand-binding (peptide-binding cavity) function remains. These strains, in combination with theoretically unlimited quantities of MAbs specific for Hwp1, Als1, and Als3 can be used to better understand dynamics of the *C. albicans* cell wall and the role of Hwp1 in host-pathogen interactions and biofilm formation.

## Data Availability Statement

The anti-Hwp1 2-E8 hybridoma was deposited in the Developmental Studies Hybridoma Bank, University of Iowa (https://dshb.biology.uiowa.edu). Other data described in this study can be found in online repositories as specified in the article and **Supplementary Material**.

## Ethics Statement

The studies involving human participants were reviewed and approved by University of Illinois at Urbana-Champaign Office for the Protection of Research Subjects. The patients/participants provided their written informed consent to participate in this study.

## Author Contributions

LH conceptualized the study. LH and ED acquired funding and were responsible for project administration. All authors developed the study methodology, performed the investigation, and conducted formal analysis. LH wrote the original draft. All authors reviewed and edited the manuscript and approved the submitted version.

## Funding

This work was supported by grant R01 DE14158 from the National Institute of Dental and Craniofacial Research, National Institutes of Health (to LH) and IDEX Transversality UFTMIP: 2016-106-CIF-D-DRDV (to HM-Y, ED).

## Conflict of Interest

The authors declare that the research was conducted in the absence of any commercial or financial relationships that could be construed as a potential conflict of interest.

## Publisher’s Note

All claims expressed in this article are solely those of the authors and do not necessarily represent those of their affiliated organizations, or those of the publisher, the editors and the reviewers. Any product that may be evaluated in this article, or claim that may be made by its manufacturer, is not guaranteed or endorsed by the publisher.
