# Supplementary material for: Development and Use of a Monoclonal Antibody Specific for the Candida albicans Cell-Surface Protein Hwp1
Source: Front Cell Infect Microbiol. 2022 Jun 27;12:907453. doi: 10.3389/fcimb.2022.907453 (PMC9273023; doi:10.3389/fcimb.2022.907453)
Supplement: Supplementary file 1 [file DataSheet_1.docx]

**SUPPLEMENTARY FILE S1 |** Repair of the *CORT_0E03570* open reading frame.

BLAST searches and information from the *Candida* Gene Order Browser (<http://cgob.ucd.ie>) identified *CORT_0E03570* as the ortholog of *C. albicans HWP1*. The *CORT_0E03570* open reading frame (ORF) was broken in the *C. orthopsilosis* reference genome sequence (chromosome 5; NC_018298) necessitating repair. Repair was accomplished by synthesizing nucleotides to amplify the broken region, followed by Sanger sequencing of the amplified PCR product. The purpose of this data sheet is to provide documentation of the broken ORF and the information used to correct it. The repaired ORF was deposited into GenBank under accession number OM802162.

The diagram below shows the map of the *C. orthopsilosis* genome where *CORT_0E03570* is located (copy/pasted from <https://www.ncbi.nlm.nih.gov/gene/14541172>).


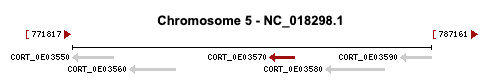


Although it had a putative stop codon (highlighted in red below), *CORT_0E03570* from the reference genome sequence was missing its start codon and over 1 kb of sequence information. A 1612-bp region was amplified using primers Co_E3570 Gap-F2 (forward; GGGATTGGTCAAATTGTGGT) and Co_E3570 Gap-R2 (reverse; GATGATCTTGATGTAGTTTCAGGC); primer sequences are highlighted in green. Sanger sequencing used these primers, as well as Co_E3570 Gap-F1 (forward; TGTTGGCACTCACATACACC) and Co_E3570 Gap-R1 (reverse; GAACTAGGTGGCTCAGACGA), highlighted in blue. The original sequence is shown, followed by the repaired sequence.

**>Original sequence from NCBI database (NC_018298)**

GGATTGGTCAAATTGTGGTGATAGTCTTTGGTATAGCATAGTTAGAATATTTAGACATTACAGAAGTACTTAGATTTCAAAAATAACGATTTTTACTGTTTAGTGAATCAGTCTGGTATTCTTATAGCTATTTAATGTTGGCACTCACATACACCAAATGCCTTTTTGTGCAAACTACTAATTATTACAGTGAGACTGCTAATACTATAGCAATACTCTATGAAAACAAGGTTTTCTCCAAAGTACGAGGACCGCAAAAATTCTCAACATTTCTGA**NNNNNNNNNNNNNNNNNNNNNNNNNNNNNNNNNNNNNNNNNNNNNNNNNNNNNNNNNNNNNNNNNNNNNNNNNNNNNNNNNNNNNNNNNNNNNNNNNNNNNNNNNNNNNNNNNNNNNNNNNNNNNNNNNNNNNNNNNNNNNNNNNNNNNNNNNNNNNNNNNNNNNNNNNNNNNNNNNNNNNNNNNNNNNNNNNNNNNNNNNNNNNNNNNNNNNNNNNNNNNNNNNNNNNNNNNNNNNNNNNNNNNNNNNNNNNNNNNNNNNNNNNNNNNNNNNNNNNNNNNNNNNNNNNNNNNNNNNNNNNNNNNNNNNNNNNNNNNNNNNNNNNNNNNNNNNNNNNNNNNNNNNNNNNNNNNNNNNNNNNNNNNNNNNNNNNNNNNNNNNNNNNNNNNNNNNNNNNNNNNNNNNNNNNNNNNNNNNNNNNNNNNNNNNNNNNNNNNNNNNNNNNNNNNNNNNNNNNNNNNNNNNNNNNNNNNNNNNNNNNNNNNNNNNNNNNNNNNNNNNNNNNNNNNNNNNNNNNNNNNNNNNNNNNNNNNNNNNNNNNNNNNNNNNNNNNNNNNNNNNNNNNNNNNNNNNNNNNNNNNNNNNNNNNNNNNNNNNNNNNNNNNNNNNNNNNNNNNNNNNNNNNNNNNNNNNNNNNNNNNNNNNNNNNNNNNNNNNNNNNNNNNNNNNNNNNNNNNNNNNNNNNNNNNNNNNNNNNNNNNNNNNNNNNNNNNNNNNNNNNNNNNNNNNNNNNNNNNNNNNNNNNNNNNNNNNNNNNNNNNNNNNNNNNNNNNNNNNNNNNNNNNNNNNNNNNNNNNNNNNNNNNNNNNNNNNNNNNNNNNNNNNNNNNNNNNNNNNNNNNNNNNNNNNNNNNNNNNNNNNNNNNNNNNNNNNNNNNNNNNNNNNNNNNNNNNNNNNNNNNNNNNNNNNNNNNNNNNNNNNNNNNNNNNNNNNNNNNNNNNNNNNNNNNNNNNNNNNNNNNNNNNNNNNNNNNNNNNNNNNNNNNNNNNNNNNNNNNNNNNNNNNNNNNNNNNNNNNNNNNNNNNNNNNNNNNNNNNNNNNNNNNNNNNNNNNNNNNNNNNNNNNNNNNNNNN**TGTGGTTGTGCACCACCCCCTCCACCACCTAGCTCACCACCACCTAGTTCTGAGCCACCTTCTTCATCAGAACCACCTAGTTCTGAACCACCAAACTCAGAGCCACCTAGTTCTGAACCACCTTCTTCGTCTGAGCCACCTAGTTCCAGACCTAGCCCACCTAGCTCGGAATCTTCATCTTCATCAAAGCCATCTAGCTCAATTGTACAACCTAGCTCTGAGAGTTTTAGTTCATCAATGCCTGAAACTACATCAAGATCATCTAGTACATCGTTGGAGTCAACAGAATCTTCTGGCCTTTCTATATCAGGAATTTCTGGGTTATCAACAGCTCGTTCCACTACGATTATTACGGTCACTACCTGCGCGCATGGAAGTTGCTCAATCATAACTGAAACTACAGGAGTGACAATTATCACTGAAGGTACTAAGATTTTTACTACTTATTGTCCATTAACAGGAGAATCATCGCCCTCTTCTTCAACTATTGGACCAAGTAATGGTTCAGGTGGCAAAGGAGGTGGAAATGGATCAAATAATGTTGGTGGCAATGGAAACGGAAGTGGATCAGGTAATGGAAACGGATCACGTTCATCAACCGTCATCGCAGTTACTACTTGCTCAAATGGCGGCTGTTCTACTGTGGTTGAAACAACTGGAGTTACAGTTAGAACAGAAGGAACAACTATATACACTACCTATTGTCCATTAACTGGAGAAACGATACCTTCATCATTAACTAGTGTTCCAAGTAATGGCTCTGGTGCTGGCAGTGGAACAGGCCAAGGGGCAGGTCAAACAGCAGGTCAAGGATCAGGAAGTGGACCAAGTCAAGGATCAGGTCCAGGAGCAGGTCAAGAGTCAATTGAGACGTCACTTACGGAGCAACAATCAACGGAGAATCATATACCATCACTACAAACGGTCTCGATTATGCAATCAAGTGAGTCTTCGTCATCAGAGTCGAATCCAGCTGAGATTTCCGTATTAGCGGCTGTTGGTCCTACGATAGGTTACTCCATTGGGGCCGTTTTCTTGGCTATTACCATGATTTTGCTTTAG

**>Repaired *CORT_0E03570* sequence**

GGATTGGTCAAATTGTGGTGATAGTCTTTGGTATAGCATAGTTAGAATATTTAGACATTACAGAAGTACTTAGATTTCAAAAATAACGATTTTTACTGTTTAGTGAATCAGTCTGGTATTCTTATAGCTATTTAATGTTGGCACTCACATACACCAAATGCCTTTTTGTGCAAACTACTAATTATTACAGTGAGACTGCTAATACTATAGCAATACTCTATGAAAACAAGGTTTTCTCCAAAGTACGAGGACCGCAAAAATTCTCAACATTTCTGA**GAATCTTAGGCGGTTGCCCATAATGAAAAGATCAATACCAAGAAAAATTTTGTTAAAAGTTTAATCTTTAAAGTGATACTGTTCTGATAGTCTACAGTCCAAATTCGATATTGCAACGAAATCACTACGGTACGAACGTATCTACGTTACTACAAACAACGATAGTCCACTTAAGAAAACGTTTTAAAAAGGTTACTGTTTACAAAATGTCCTTTTCTGGGAGTCACTTTGATCAGAAATGATCCTTTTACGGAATTGAGTAAATGCTACCTGGGAGGTATTACCGGGCATCTATTAGCCTTATTTATGATGAATTTCATATCTCGCATGACTTTGGTTTGCAACAAGGTTAGTTTTGCTTTGCTTCAGGATCAGCACAGAATTCCAAATAATGAGGTTTCACCATGGAATAAAGCAAGGAGGACAACACCCTACCATTACAGCCTCCCCTATGTGACGTGGACAAGTTATATTGCGACAACACATTCAACCCTCAGTACTACATTACCACTTTGAATGCGGCGTATGTAGTTTCAGACTCAGAGAGAAATGTTGATAGCAGCTACAGCATCATCATTAACTACCAAGCCGATAAATATGATCGGTTGTATGACAATTTTGCATCAAATATTGACCAAATATACCTATCAGACACGGAGTCGCAGACCCAATTCTCTATAACTACCTATTACCAAACACCATCACCAACCCATTTCGCTGGTCTGCCAAGATTCAGGTCAGACCACAACTAAAGAATGGTAAGTGTTGCATTTCGGATTCGTTTAGACTCTGGTATAGATTTAGAATAATCGGTACTGGTGGTGCAGCGATAAACCAAGTTTTTGGACAGCCATTCAGTTACTACAGCACCAACGTATGGTCGGGACCGGTTCAACAGTATGATCCAATGACTTTGTTCAATCAACAGTTGATGTTTCAAAAAAGAGACCTGGAGGACAAAGGTGATGCGGGTGAGTTGGATACTTTAGATAAAAGAAGCTATCAGTCAATGGTTGAATTATTGAATAGCTGCACCACGAAACATCATGGTATCAAGCAATTTTGTTGGGAC**TGTGGTTGTGCACCACCCCCTCCACCACCTAGCTCACCACCACCTAGTTCTGAGCCACCTTCTTCATCAGAACCACCTAGTTCTGAACCACCAAACTCAGAGCCACCTAGTTCTGAACCACCTTCTTCGTCTGAGCCACCTAGTTCCAGACCTAGCCCACCTAGCTCGGAATCTTCATCTTCATCAAAGCCATCTAGCTCAATTGTACAACCTAGCTCTGAGAGTTTTAGTTCATCAATGCCTGAAACTACATCAAGATCATCTAGTACATCGTTGGAGTCAACAGAATCTTCTGGCCTTTCTATATCAGGAATTTCTGGGTTATCAACAGCTCGTTCCACTACGATTATTACGGTCACTACCTGCGCGCATGGAAGTTGCTCAATCATAACTGAAACTACAGGAGTGACAATTATCACTGAAGGTACTAAGATTTTTACTACTTATTGTCCATTAACAGGAGAATCATCGCCCTCTTCTTCAACTATTGGACCAAGTAATGGTTCAGGTGGCAAAGGAGGTGGAAATGGATCAAATAATGTTGGTGGCAATGGAAACGGAAGTGGATCAGGTAATGGAAACGGATCACGTTCATCAACCGTCATCGCAGTTACTACTTGCTCAAATGGCGGCTGTTCTACTGTGGTTGAAACAACTGGAGTTACAGTTAGAACAGAAGGAACAACTATATACACTACCTATTGTCCATTAACTGGAGAAACGATACCTTCATCATTAACTAGTGTTCCAAGTAATGGCTCTGGTGCTGGCAGTGGAACAGGCCAAGGGGCAGGTCAAACAGCAGGTCAAGGATCAGGAAGTGGACCAAGTCAAGGATCAGGTCCAGGAGCAGGTCAAGAGTCAATTGAGACGTCACTTACGGAGCAACAATCAACGGAGAATCATATACCATCACTACAAACGGTCTCGATTATGCAATCAAGTGAGTCTTCGTCATCAGAGTCGAATCCAGCTGAGATTTCCGTATTAGCGGCTGTTGGTCCTACGATAGGTTACTCCATTGGGGCCGTTTTCTTGGCTATTACCATGATTTTGCTTTAG

The protein predicted from the corrected *CORT_0E03570* does not have an apparent signal peptide processing site. The putative start codon (highlighted in green above) is the only one that predicts a sequence similar to the other *C. albicans* Hwp1 orthologs. The predicted protein has a putative GPI anchor addition site with the best matching cleavage site highlighted in red below. A sequence alignment with *C. albicans* Hwp1, created using Clustal Omega (<https://www.ebi.ac.uk/Tools/msa/clustalo>), is also shown.

**>CORT_0E03570 = Predicted protein from repaired *C. orthopsilosis* ORF**

MTLFNQQLMFQKRDSEDKGDAGELDTLDKRSYQSMVELLNSCTTKHHGIKQFCWDCGCAPPPPPPSSPPPSSEPPSSSEPPSSEPPNSEPPSSEPPSSSEPPSSRPSPPSSESSSSSKPSSSIVQPSSESFSSSMPETTSRSSSTSLESTESSGLSISGISGLSTARSTTIITVTTCAHGSCSIITETTGVTIITEGTKIFTTYCPLTGESSPSSSTIGPSNGSGGKGGGNGSNNVGGNGNGSGSGNGNGSRSSTVIAVTTCSNGGCSTVVETTGVTVRTEGTTIYTTYCPLTGETIPSSLTSVPSNGSGAGSGTGQGAGQTAGQGSGSGPSQGSGPGAGQESIETSLTEQQSTENHIPSLQTVSIMQSSESSSSESNPAEISVLA**A**VGPTIGYSIGAVFLAITMILL

**>C4_03570W_A = orf19.1321 = *C. albicans* Hwp1**

**MRLSTAQLIAIAYYMLSIGATVPQVDG**QGETEEALIQKRSYDYYQEPCDDYPQQQQQQEPCDYPQQQQQEEPCDYPQQQPQEPCDYPQQPQEPCDYPQQPQEPCDYPQQPQEPCDNPPQPDVPCDNPPQPDVPCDNPPQPDVPCDNPPQPDVPCDNPPQPDQPDDNPPIPNIPTDWIPNIPTDWIPDIPEKPTTPATTPNIPATTTTSESSSSSSSSSSSTTPKTSASTTPESSVPATTPNTSVPTTSSESTTPATSPESSVPVTSGSSILATTSESSSAPATTPNTSVPTTTTEAKSSSTPLTTTTEHDTTVVTVTSCSNSVCTESEVTTGVIVITSKDTIYTTYCPLTETTPVSTAPATETPTGTVSTSTEQSTTVITVTSCSESSCTESEVTTGVVVVTSEETVYTTFCPLTENTPGTDSTPEASIPPMETIPAGSEPSMPAGETSPAVPKSDVPATESAPVPEMTPAGSQPSIPAGETSPAVPKSDVSATESAPAPEMTPAGTETKPAAPKSSAPATEPSPVAPGTESAPAGPGASSSPKSSVLASETSPIAPGAETAPAGSSGAITIPESSAVVSTTEGAIPTTLESVPLMQPSANYSSVAPISTFE**G**AGNNMRLTFGAAIIGIAAFLI

CORT_0E03570 MTLFNQQLM------F----QKRDSEDKGDAGELDTLDKRSYQSMVELL----------- 39

orf19.1321 MRLSTAQLIAIAYYMLSIGATVPQVDGQGE-TEEALIQKRSYDYYQEPCDDYPQQQQQQE 59

* * . **: : : :.:*: * ::****: *

CORT_0E03570 -----------NSCTTKHHGIKQFC---------------------------WDCGCAPP 61

orf19.1321 PCDYPQQQQQEEPCDYPQQQPQEPCDYPQQPQEPCDYPQQPQEPCDYPQQPQEPCDNPPQ 119

: * :: :: * *. *

CORT_0E03570 PPPPSSPPPSSE-----PP-----S----SSEPPSSEPPNSEPPSSEPPSSSEP------ 101

orf19.1321 PDVPCDNPPQPDVPCDNPPQPDVPCDNPPQPDVPCDNPPQPDQPDDNPPIPNIPTDWIPN 179

* *.. **. : ** . . : *..:**: : *..:** . *

CORT_0E03570 ---------------PSSRPSPPS----SESSSSSKPSSSIVQPSS---ESFSSSMPE-- 137

orf19.1321 IPTDWIPDIPEKPTTPATTPNIPATTTTSESSSSSSSSSSSTTPKTSASTTPESSVPATT 239

*:: *. *: *******. *** . *.: : .**:*

CORT_0E03570 -------TTSR--------------------------SSS-------TSLESTESSGLSI 157

orf19.1321 PNTSVPTTSSESTTPATSPESSVPVTSGSSILATTSESSSAPATTPNTSVPTTTTEAKSS 299

*:*. *** **: :* :.. *

CORT_0E03570 SGISGLSTARSTTIITVTTCAHGSCSIITETTGVTIITEGTKIFTTYCPLTGESSPSSST 217

orf19.1321 STPLTTTTEHDTTVVTVTSCSNSVCTESEVTTGVIVITSKDTIYTTYCPLTETTPVSTAP 359

* :* :.**::***:*::. *: **** :**. .*:******* : *::

CORT_0E03570 IGPSNGSGGKGGGNGSNNVGGNGNGSGSGNGNGSRSSTVIAVTTCSNGGCSTVVETTGVT 277

orf19.1321 AT--------------------ETPTGTVSTSTEQSTTVITVTSCSESSCTESEVTTGVV 399

. :*: . . .:*:***:**:**:..*: ****.

CORT_0E03570 VRTEGTTIYTTYCPLTGETIPSSLTSV---P-SNGSGAGSGTGQGAGQTAGQ-------- 325

orf19.1321 VVTSEETVYTTFCPLTENTPGTDSTPEASIPPMETIPAGSEPSMPAGETSPAVPKSDVPA 459

* *. *:***:**** :* :. * * : *** . **:*:

CORT_0E03570 ----------GSGSGPSQ------------------------------------------ 333

orf19.1321 TESAPVPEMTPAGSQPSIPAGETSPAVPKSDVSATESAPAPEMTPAGTETKPAAPKSSAP 519

:** **

CORT_0E03570 --------------GSGPGAGQESIETSLTE----------------------------Q 351

orf19.1321 ATEPSPVAPGTESAPAGPGASSSPKSSVLASETSPIAPGAETAPAGSSGAITIPESSAVV 579

:****... .: *:.

CORT_0E03570 QSTENHIP-SLQTVSIMQSSESSSSESNPAEISVLAAVGPTIGYSIGAVFLAITMILL 408

orf19.1321 STTEGAIPTTLESVPLMQPSA---NYSSVAPISTFEGAGNNMRLTFGAAIIGIAAFLI 634

.:**. ** :*::* :** * . *. * **.: ..* .: ::**.::.*: :*:
