# Supplementary material for: Development and Use of a Monoclonal Antibody Specific for the Candida albicans Cell-Surface Protein Hwp1
Source: Front Cell Infect Microbiol. 2022 Jun 27;12:907453. doi: 10.3389/fcimb.2022.907453 (PMC9273023; doi:10.3389/fcimb.2022.907453)
Supplement: Supplementary file 2 [file DataSheet_2.docx]

**SUPPLEMENTARY FILE S2 |** Repair of the *CORT_0E03560* open reading frame.

BLAST searches and information from the *Candida* Gene Order Browser (<http://cgob.ucd.ie>) identified *CORT_0E03560* as the ortholog of *C. albicans RBT1*. The *CORT_0E03560* open reading frame (ORF) was broken in the *C. orthopsilosis* reference genome sequence (chromosome 5; NC_018298) necessitating repair. Repair was accomplished by synthesizing nucleotides to amplify the broken region, followed by Sanger sequencing of the amplified PCR product. The purpose of this data sheet is to provide documentation of the broken ORF and the information used to correct it. The repaired ORF was deposited into GenBank under accession number MZ509453.

The diagram below shows the map of the *C. orthopsilosis* genome where *CORT_0E03560* is located (copy/pasted from <https://www.ncbi.nlm.nih.gov/gene/?term=CORT_0E03560>).


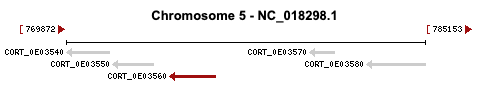


Although it had putative start and stop codons (highlighted in green and red, respectively, below), *CORT_0E03560* from the reference genome sequence had a gap of unknown sequence (NNN highlighted in yellow) in the center of the coding region. A 2569-bp region was amplified using primers Co_E3560 NT-F1 (forward; ACAATGATTGAAGGCGGTTG) and Co_E3560 CT-R1 (reverse; GAACTTGCCCGACTTGC); primer sequences are highlighted in green. Sanger sequencing used these primers, as well as Co_E3560 NT-Seq-F1 (forward; GTTTCGAGTGCTTTCAAGTGG), Co_E3560 Gap-R1 (reverse; CAACAGTTTCTGTTGAGCCA), and Co_E3560 CT-Seq-R1 (reverse; TGATTCTGGCCCTGATTCG), highlighted in blue. The original sequence is shown, followed by the corrected sequence. The 401 nucleotides that were missing in the reference genome sequence are highlighted in gray. No other differences were noted between the reference and repaired sequences.

**>Original sequence from NCBI database (NC_018298)**

ATGAAGTTATCCACTGCCAACTTATTGTCACTCGCTGCATTTGTGTCTTCAACTCAAGCCACCTGGTCTTTATTTGAAGATTTATTCAAAGATTGTGCTCCAAAGCACGTTCAACCTATCTGTGAGATTCAGGATATCTTTTGTGACACTGATGATTCGAATGACTCAGTTTCTGCTCGTGATGCTTCTGTTAACTTTAAAGCTGCTTTTGTTGTTTCTGGTGCTGAAAAAAATGATGATGGAACTTATAACGTTGTTGCGAATTATGAAGCTGATCAATCTGACCAATTGCATCAGTACTTTGGTGGTAACATTGACCTGTTGTCTCTTACTGGTACTGGTTGTGATGATGTTCAGCTTTATGGACAAGGTGCTTCCAATGCTGTTTCGAGTGCTTTCAAGTGGTCTACAAAATTTCAATGTAAACCAGAATACAAAAATGGAAAATGTTGTCTTCCAGACGGTTTTACAATTGGGTTCAAATTTATTGAGATTGGTCTTGCTTGGGAAGCATTAAAGTTGGTTTTTGGTCATCAATCGTGTCTGTATGGTATTATTACTCATTGGAGCTTTTTGGAGGTTTTTGATCCATCGGCATTGCTTCATCATGATTTATCATTATTTAAGAGAGACGAAGTTGCTGATGGTGTTGAAGGATTCACCACTTTTGATAAGAGAACCTTGGGTTTCTTGCATGACTTGTTAGGTAAATGTACTACTCAAAACAAGGGAATTAAACAGTTCTGTTGGGAATGCGACTGTCCATCTACATCGAGTAGCACTACTGTTCCACCAAGCACTTCATCTACTCCTCCAACTGAGTCGTCTACTCCTCCAACTGAATCATCTACTCCACCAACTGAATCGTGTACTCCACCAACTGAGTCGTCTACTCCACCAACTGAGTCGTCTACTCCACCAACCGAGTCTTCTACTCCTCCAACTGAGTCGTCTACTCCACCAACTGAATCATCTACTCCTCCAACTGAATCATCTACTCCTCCAACCGAGTCTTCAACTCCACCAACTGAGTCGTCTA**NNNNNNNNNNNNNNNNNNNNNNNNNN**CCAACTGAGTCTTCAACTCCTCCAACTGAATCATCTACTCCTCCAACCGAGTCTTCTACTCCTCCAACTGAGTCGTCTACTCCACCAACTGAATCATCTACTCCTCCAACTGAATCATCTACTCCTCCAACTGAGTCGTATACTTCTTCTGGCTCAACAGAAACTGTTGTTACCCACTCTACAACCATTGTTACTATTACCTCTTGTTCTGAAGATAAATGTGAAACTACAACAGCCACCACTGGAATCACTGTCATTACTGAAGGAACTACAATTTACACAACGTACTGTCCTTTGACTGAAAAGACTACTTCGTCAGCAACAACTTCCAAGGAAACATCTGTACCAGAAACAGTCTCCACCATTACTACAGCTTCCACTGGACCAACTGAGAGTAACGAATCAGGGCCAGAATCAAGTACAAGGGTACCAACAACAACAACCGCTACTGCTCCTTCTACTTCTGTCCCGGTTAGCTCGCAACCCCCATCAAAGAGTCAAAGTGAAGCTCCAAAATCAGAAGGTGAATCAGAAACTGCTGCTGCTCCAACTAGTGAAAGTGAAGCGCCAAAATCAGAAGGTGAATCAGAAACTGCTGCTGCTCCTTCTTCATCTGCTCCTGCCGGTCAAAGCGAAGCCCCATCTTCTGCTCCAGGTGAATCACAAACTGTTGTGTCTCCTTCTTCTGCTCCAGCTTCCTCGCAAGCCCCTTCAGAGGACCAAGGTCAAGCCCCAGCATCCGAAGGTGAATCACAAACGGTAGAAGCTCAATCATCATCTACTCCAAAGGTTTCACAAGCTCCATCAGGAGGTGAGAATGAAACCACTGCTGCTATTTCTACATATGAGGGTGCCGCTATGAGAAATTCAGCAGGAATTCTTTTGGTTGGTGCTGCTGCGTTGTTACTTTAA

**>Repaired *CORT_0E03560* sequence**

ACAATGATTGAAGGCGGTTGTAATATCTTTGCAATCTAACAATGAAGAATATATAAAGGGGTGAATATTTCACCATATTTGAGGTCATTCAAAAGATTCTTAACCCAACAAAGCTCAGGTAATCACTATAACAATGAAGTTATCCACTGCCAACTTATTGTCACTCGCTGCATTTGTGTCTTCAACTCAAGCCACCTGGTCTTTATTTGAAGATTTATTCAAAGATTGTGCTCCAAAGCACGTTCAACCTATCTGTGAGATTCAGGATATCTTTTGTGACACTGATGATTCGAATGACTCAGTTTCTGCTCGTGATGCTTCTGTTAACTTTAAAGCTGCTTTTGTTGTTTCTGGTGCTGAAAAAAATGATGATGGAACTTATAACGTTGTTGCGAATTATGAAGCTGATCAATCTGACCAATTGCATCAGTACTTTGGTGGTAACATTGACCTGTTGTCTCTTACTGGTACTGGTTGTGATGATGTTCAGCTTTATGGACAAGGTGCTTCCAATGCTGTTTCGAGTGCTTTCAAGTGGTCTACAAAATTTCAATGTAAACCAGAATACAAAAATGGAAAATGTTGTCTTCCAGACGGTTTTACAATTGGGTTCAAATTTATTGAGATTGGTCTTGCTTGGGAAGCATTAAAGTTGGTTTTTGGTCATCAATCGTGTCTGTATGGTATTATTACTCATTGGAGCTTTTTGGAGGTTTTTGATCCATCGGCATTGCTTCATCATGATTTATCATTATTTAAGAGAGACGAAGTTGCTGATGGTGTTGAAGGATTCACCACTTTTGATAAGAGAACCTTGGGTTTCTTGCATGACTTGTTAGGTAAATGTACTACTCAAAACAAGGGAATTAAACAGTTCTGTTGGGAATGCGACTGTCCATCTACATCGAGTAGCACTACTGTTCCACCAAGCACTTCATCTACTCCTCCAACTGAGTCGTCTACTCCTCCAACTGAATCATCTACTCCACCAACTGAATCGTGTACTCCACCAACTGAGTCGTCTACTCCACCAACTGAGTCGTCTACTCCACCAACCGAGTCTTCTACTCCTCCAACTGAGTCGTCTACTCCACCAACTGAATCATCTACTCCTCCAACTGAATCATCTACTCCTCCAACCGAGTCTTCAACTCCACCAACTGAGTCGTCTA**CTCCACCAACTGAGTCGTCTACTCCACCAACCGAGTCTTCAACTCCACCAACTGAGTCGTCTACTCCTCCAACTGAGTCGTCTACTCCACCAACCGAGTCTTCAACTCCACCAACTGAGTCTTCAACTCCACCAACTGAGTCGTCTACTCCTCCAACCGAGTCTTCAACTCCACCAACTGAGTCGTCTACTCCACCAACTGAGTCGTCTACTCCACCAACCGAGTCTTCAACTCCACCAACTGAGTCGTCTACTCCACCAACCGAGTCTTCAACTCCACCAACTGAGTCGTCTACTCCTCCAACCGAGTCTTCAACTCCTCCAACCGAGTCTTCAACTCCTCCAACTGAGTCTTCAACTCCACCAACTGAGTCGTCTACTCCTCCAACTGAGTCTTCAACT**CCTCCAACTGAATCATCTACTCCTCCAACCGAGTCTTCTACTCCTCCAACTGAGTCGTCTACTCCACCAACTGAATCATCTACTCCTCCAACTGAATCATCTACTCCTCCAACTGAGTCGTATACTTCTTCTGGCTCAACAGAAACTGTTGTTACCCACTCTACAACCATTGTTACTATTACCTCTTGTTCTGAAGATAAATGTGAAACTACAACAGCCACCACTGGAATCACTGTCATTACTGAAGGAACTACAATTTACACAACGTACTGTCCTTTGACTGAAAAGACTACTTCGTCAGCAACAACTTCCAAGGAAACATCTGTACCAGAAACAGTCTCCACCATTACTACAGCTTCCACTGGACCAACTGAGAGTAACGAATCAGGGCCAGAATCAAGTACAAGGGTACCAACAACAACAACCGCTACTGCTCCTTCTACTTCTGTCCCGGTTAGCTCGCAACCCCCATCAAAGAGTCAAAGTGAAGCTCCAAAATCAGAAGGTGAATCAGAAACTGCTGCTGCTCCAACTAGTGAAAGTGAAGCGCCAAAATCAGAAGGTGAATCAGAAACTGCTGCTGCTCCTTCTTCATCTGCTCCTGCCGGTCAAAGCGAAGCCCCATCTTCTGCTCCAGGTGAATCACAAACTGTTGTGTCTCCTTCTTCTGCTCCAGCTTCCTCGCAAGCCCCTTCAGAGGACCAAGGTCAAGCCCCAGCATCCGAAGGTGAATCACAAACGGTAGAAGCTCAATCATCATCTACTCCAAAGGTTTCACAAGCTCCATCAGGAGGTGAGAATGAAACCACTGCTGCTATTTCTACATATGAGGGTGCCGCTATGAGAAATTCAGCAGGAATTCTTTTGGTTGGTGCTGCTGCGTTGTTACTTTAATCTTAATCATAAAATAGTCTTGATACTTTTACTGTTCTATCATTTGATACCATAATTTCGAGGAAAACTCACCATCTAGATGATGGCAAGTCGGGCAAGTTC

The protein predicted from the corrected *CORT_0E03560* has a putative signal peptide processing site (bold black type). The predicted protein has a putative GPI anchor addition site with the best matching cleavage site highlighted in red below. A sequence alignment with *C. albicans* Rbt1, created using Clustal Omega (<https://www.ebi.ac.uk/Tools/msa/clustalo>), is also shown.

**>CORT_0E03560 = Predicted protein from repaired *C. orthopsilosis* ORF**

**MKLSTANLLSLAAFVSSTQA**TWSLFEDLFKDCAPKHVQPICEIQDIFCDTDDSNDSVSARDASVNFKAAFVVSGAEKNDDGTYNVVANYEADQSDQLHQYFGGNIDSLSLTGTGCDDVQLYGQGASNAVSSAFKWSTKFQCKPEYKNGKCCLPDGFTIGFKFIEIGLAWEALKLVFGHQSCSYGIITHWSFLEVFDPSALLHHDLSLFKRDEVADGVEGFTTFDKRTLGFLHDLLGKCTTQNKGIKQFCWECDCPSTSSSTTVPPSTSSTPPTESSTPPTESSTPPTESCTPPTESSTPPTESSTPPTESSTPPTESSTPPTESSTPPTESSTPPTESSTPPTESSTPPTESSTPPTESSTPPTESSTPPTESSTPPTESSTPPTESSTPPTESSTPPTESSTPPTESSTPPTESSTPPTESSTPPTESSTPPTESSTPPTESSTPPTESSTPPTESSTPPTESSTPPTESSTPPTESSTPPTESSTPPTESSTPPTESSTPPTESSTPPTESSTPPTESYTSSGSTETVVTHSTTIVTITSCSEDKCETTTATTGITVITEGTTIYTTYCPLTEKTTSSATTSKETSVPETVSTITTASTGPTESNESGPESSTRVPTTTTATAPSTSVPVSSQPPSKSQSEAPKSEGESETAAAPTSESEAPKSEGESETAAAPSSSAPAGQSEAPSSAPGESQTVVSPSSAPASSQAPSEDQGQAPASEGESQTVEAQSSSTPKVSQAPSGGENETTAAISTYE**G**AAMRNSAGILLVGAAALLL

**>C4_03520C_A = orf19.1327 = *C. albicans* Rbt1**

**MRFATAQLAALAYYILSTEA**TFPLLGDIFNCIPHNTPPVCTDLGLYHDSSISLGGSKNKREAEIANKDGTIEKRTFGSAGVNAGFNAAFVVSNAKKLSDGSYGIDCNFKSDSSVQLNSAFGKKVKQLSITGTGYSDISLLGNVANPFEWSASLKVKAEIVKGKCCLPSGFRIVTDFESNCPEFDAIKQFFGSSQIIYKVNAVSNAIGTFDASALFNAQVKAFPAKRELDEFEELSNDGVTHSKRTLGLLLGLLKKVTGGCDTLQQFCWDCQCDTPSPSTTTVSTSSAPSTSPESSAPSTTTVTTSSSPVTSPESSVPETTTVTTSSVPETTPESSAPETTTVTTSSVPSTTPESSAPETTPESSAPESSVPESSAPETTPESSAPESSVPESSAPETETETTPTAHLTTTTAQTTTVITVTSCSNNACSKTEVTTGVVVVTSEDTIYTTFCPLTETTPVPSSVDSTSVTSAPETTPESTAPESSAPESSAPESSAPVTETPTGPVSTVTEQSKTIVTITSCSNNACSESKVTTGVVVVTSEDTVYTTFCPLTETTPATESASESSAPATESVPATESAPVAPESSAPGTETAPATESAPATESSPVAPGTETTPATPGAESTPVTPVAPESSAPAVESSPVAPGVETTPVAPVAPSTTAKTSALVSTTEGTIPTTLESVPAIQPSANSSYTIASVSSFE**G**AGNNMRLTYGAAIIGLAAFLI

CORT_0E03560 MKLSTANLLSLAAFVSSTQATWSLFEDLFKDCAPKHVQPICEIQDIFCDTDDSND----- 55

orf19.1327 MRFATAQLAALAYYILSTEATFPLLGDIF-NCIPHNTPPVCTDLGLYHDSSISLGGSKNK 59

*:::**:* :** :: **:**: *: *:* :* *::. *:* .:: *:. * .

CORT_0E03560 ---------------SVSARDASVNFKAAFVVSGAEKNDDGTYNVVANYEADQSDQLHQY 100

orf19.1327 REAEIANKDGTIEKRTFGSAGVNAGFNAAFVVSNAKKLSDGSYGIDCNFKSDSSVQLNSA 119

:..: .....*:******.*:* .**:*.: .*:::*.* **:.

CORT_0E03560 FGGNIDSLSLTGTGCDDVQLYGQGASNAVSSAFKWSTKFQCKPEYKNGKCCLPDGFTIGF 160

orf19.1327 FGKKVKQLSITGTGYSDISLLGN-----VANPFEWSASLKVKAEIVKGKCCLPSGFRIVT 174

** ::..**:**** .*:.* *: *:. *:**:.:: * * :******.** *

CORT_0E03560 KFIEIGLAWEALKLVFGHQSCSYGIITHWSFLEVFDPSALLHHDLSLFKRDEVADGVEG- 219

orf19.1327 DFESNCPEFDAIKQFFGSSQIIYKVNAVSNAIGTFDASALFNAQVKAFPAKRELDEFEEL 234

.* . ::*:* .** .. * : : . : .** ***:: ::. * .. * .*

CORT_0E03560 ---FTTFDKRTLGFLHDLLGKCTTQNKGIKQFCWECDCPSTSSSTTVPPSTSSTP-PTES 275

orf19.1327 SNDGVTHSKRTLGLLLGLLKKVTGGCDTLQQFCWDCQCDTPSPSTTTVSTSSAPSTSPES 294

.*..*****:* .** * * . ::****:*:* : * ***. ::*: **

CORT_0E03560 STPPTESSTPPTESCTPPTESSTPPTESSTPPTESSTPPTESSTPPTESSTPPTESSTPP 335

orf19.1327 SAPSTTTVTT---SSSPV----TS-PESSVPETTTV---TTSSVPETT----P-ESSAPE 338

*:* * : * *.:* * ***.* * : * **.* * * ***:*

CORT_0E03560 TESSTPPTESSTPPTESSTPPTESSTPPTESSTPPTESSTPPTESSTPPTESSTPPTESS 395

orf19.1327 TTTV---TTSSVPST---T-P-ESSAPETTPESSAPESSVPE--SSAPETTPESSAPESS 388

* : * **.* * * * ***:* * .: ***.* **:* * .: ***

CORT_0E03560 TPPTESSTPPTESSTPPTESSTPPTESST-PPTESSTPPTESSTPPTESSTPPTESSTPP 454

orf19.1327 VP--ESSAPETETETTPTAHLTTTTAQTTTVITVTSCS--NNACSKTEVTTGVV---VVT 441

.* ***:* **:.* ** * * .:* * :* :.: ** :* . .

CORT_0E03560 TESSTPPTESSTPPTESSTPPTESSTPPTESSTPPTESSTPPTESSTPPTESSTPPTESS 514

orf19.1327 SEDT--IYTTFCPLTETTPVPSSV-DSTSVTSAPETTPESTAPESSAP--ESSA--PESS 494

:*.: : * **:: *:. : :*:* * .: ***:* ***: ***

CORT_0E03560 TPPTESYTSSGSTETVVTHSTTIVTITSCSEDKCETTTATTGITVITEGTTIYTTYCPLT 574

orf19.1327 APV--TETPTGPVSTVTEQSKTIVTITSCSNNACSESKVTTGVVVVTSEDTVYTTFCPLT 552

:* : * :* ..**. :*.*********:: *. :..***:.*:*. *:***:****

CORT_0E03560 EKTTSSATTSKETSVPETVSTITTASTGPTESNESGPESSTRVPTTTTATAPSTSVPVSS 634

orf19.1327 ETTPATESASES-SAPATESVP------ATESAPVAPESSA--PGTE--TAPAT----ES 597

*.* :: ::*:. *.* * *. *** .****: * * ***:* .*

CORT_0E03560 QPPSKSQSEAPKSEGESETAAAPTSESEAPKSEGESETAAAPSSSAPAGQSEAPSSAPGE 694

orf19.1327 APATES---SPVAPGTETTPATPG-------AESTPVTPVAPESSAPAVES--SPVAPGV 645

* ::* :* : * . * *:* :*. * .**.***** :* ***

CORT_0E03560 SQTVVSPSS---APASSQAPSEDQGQAPASEGESQTVEAQSSSTPKVSQAPSGGENETTA 751

orf19.1327 ETTPVAPVAPSTTAKTSALVSTTEGTIPTT----------LESVPA--IQPSANSSYTIA 693

. * *:* : : :* * :* *:: .*.* **.... * *

CORT_0E03560 AISTYEG--AAMRNSAGILLVGAAALLL 777

orf19.1327 SVSSFEGAGNNMRLTYGAAIIGLAAFLI 721

::*::** ** : * ::* **:*:
