# Supplementary material for: Development and Use of a Monoclonal Antibody Specific for the Candida albicans Cell-Surface Protein Hwp1
Source: Front Cell Infect Microbiol. 2022 Jun 27;12:907453. doi: 10.3389/fcimb.2022.907453 (PMC9273023; doi:10.3389/fcimb.2022.907453)
Supplement: Supplementary file 3 [file DataSheet_3.docx]

**SUPPLEMENTARY FILE S3 |** Repair of the *CORT_0E05950* open reading frame.

BLAST searches identified *CORT_0E05950* as sharing sequence similarity with *C. albicans RBT1*. The *CORT_0E05950* open reading frame (ORF) stopped prematurely in the *C. orthopsilosis* reference genome sequence (chromosome 5; NC_018298) necessitating repair. Repair was accomplished by synthesizing nucleotides to amplify the broken region, followed by Sanger sequencing of the amplified PCR product. The purpose of this data sheet is to provide documentation of the broken ORF and the information used to correct it. The repaired ORF was deposited into GenBank under accession number MZ509454.

The diagram below shows the map of the *C. orthopsilosis* genome where *CORT_0E05950* is located (copy/pasted from <https://www.ncbi.nlm.nih.gov/gene/14540888>).

**Chromosome 5 - NC_018298.1**
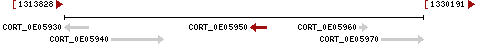


Sequence alignments from BLAST results and available genome information suggested that *CO_0E05950* had the correct start codon (highlighted in green) but stopped prematurely, yielding a predicted protein that was far too short.

Although it had a putative stop codon (highlighted in red below), *CORT_0E03570* from the reference genome sequence was missing its start codon and over 1 kb of sequence information. A 3072-bp region was amplified using primers Co_E05950 NT-F1 (forward; ATGTACCCAAGCATTTACGAGAG) and Co_E05950 CT-R1 (reverse; ATGACTGTTGAGAGATAAGAAGGC); primer sequences are highlighted in green. Sanger sequencing used these primers, as well as Co_E05950 NT-Seq-F1 (forward; GGGACGCTCAACTATATGATCC), Co_E5950 Gap-F1 (forward; AGGACCTCAAATGGGTTGC), Co_E05950 CT-Seq-R1 (reverse; AAACCGGAACCAGCTCC), and Co_E5950 Gap-R1 (reverse; CTGATCCGCTTCCTGACC) highlighted in blue. The original sequence is shown, followed by the repaired sequence.

**>Original sequence from NCBI database (NC_018298)**

ATGAGACTTTCAACAATCCAGTTGTTAGTCTATTTTGTGTGCTCAATACAAGCCTCCCTGGTTTTCTTTCCAAATTCATTCAATGATTGCACCCCACCAACAAAACCTGTCTGCACGGTAAATGAAGTTTACTGCGACAACAACTCCAACAATCCACAATATAGTAACGCCAATTTTAATGCCGCTTTTGTTGTGTCTGAAGCTGAAAAGAATGCTGATGGTAGTTATAATGTTGTTGGTAATTATCAAGCTGCTCAATCAGACCAATTGTTTAACAATTTTGCACAGAACGTTGATCAAATCTACATTAGCGGAACAGGTGTCAATGATGCAACTATTTACAGTCAATCAACCAACAACCCAGTCAACAATCCTTTCAAATGGTCAGCCAAATTTCGCTGTCAACCGCAGATTAAATTTGGCAAGTGCTGTATTCCGGATGGTTTGAAACTCTACTATAAATTTAAACAAGGTGGTGCAGGGGGTGCAGTGTTGAATACGGTTTTTGGACAACAACAACTAAGCTACTACAATACCAACCCTTTATCTGGGGACGCTCAACTATATGATCCCAACAATTTGTTCAATCAACAATTGTCGTTTCACAAGAGAGACGAAGAAGCTGATGTCGACCAGAGAACTGAATTGAGTAAAAGGACCTCAAATGGGTTGCTCAATTTATTGATTAGCTGCACTAATAAGCACCAAGGAATTAAGCAATTCTGTTGGGATTGTG**NNNNNNNNNNNNNNNNNNNNNNNNNNNNNNNNNNNNNNNNNNNNNNNNNNNNNNNNNNNNNNNNNNNNNNNNNNNNNNNNNNNNNNNNNNNNNNNNNNNNNNNNNNNNNNNNNNNNNNNNNNNNNNNNNNNNNNNNNNNNNNNNNNNNNNNNNNNNNNNNNNNNNNNNNNNNNNNNNNNNNNNNNNNNNNNNNNNNNNNNNNNNNNNNNNNNNNNNNNNNNNNNNNNNNNNNNNNNNNNNNNNNNNNNNNNNNNNNNNNNNNNNNNNNNNNNNNNNNNNNNNNNNNNNNNNNNNNNNNNNNNNNNNNNNNNNNNNNNNNNNNNNNNNNNNNNNNNNNNNNNNNNNNNNNNNNNNNNNNNNNNNNNNNNNNNNNNNNNNNNNNNNNNNNNNNNNNNNNNNNNNNNNNNNNNNNNNNNNNNNNNNNNNNNNNNNNNNNNNNNNNNNNNNNNNNNNNNNNNNNNNNNNNNNNNNNNNNNNNNNNNNNNNNNNNNNNNNNNNNNNNNNNNNNNNNNNNNNNNNNNNNNNNNNNNNNNNNNNNNNNNNNNNNNNNNNNNNNNNNNNNNNNNNNNNNNNNNNNNNNNNNNNNNNNNNNNNNNNNNNNNNNNNNNNNNNNNNNNNNNNNNNNNNNNNNNNNNNNNNNNNNNNNNNNNNNNNNNNNNNNNNNNNNNNNNNNNNNNNNNNNNNNNNNNNNNNNNNNNNNNNNNNNNNNNNNNNNNNNNNNNNNNNNNNNNNNNNNNNNNNNNNNNNNNNNNNNNNNNNNNNNNNNNNNNNNNNNNNNNNNNNNNNNNNNNNNNNNNNNNNNNNNNNNNNNNNNNNNNNNNNNNNNNNNNNNNNNNNNNNNNNNNNNNNNNNNNNNNNNNNNNNNNNNNNNNNNNNNNNNNNNNNNNNNNNNNNNNNNNNNNNNNNNNNNNNNNNNNNNNNNNNNNNNNNNNNNNNNNNNNNNNNNNNNNNNNNNNNNNNNNNNNNNNNNNNNNNNNNNNNNNNNNNNNNNNNNNNNNNNNNNNNNNNNNNNNNNNNNNNNNNNNNNNNNNNNNNNNNNNNNNNNNNNNNNNNNNNNNNNNNNNNNNNNNNNNNNNNNNNNNNNNNNNNNNNNNNNNNNNNNNNNNNNNNNNNNNNNNNNNNNNNNNNNNNNNNNNNNN**GGATCAGGAAGTGGATCAGGAAGTGGATCA**G**AAG**GTA**AT**A**A**T**G**GGTCGGGT**AATGGGTCGGGTAATGGATCAGGAAGTGG**G**TCAGGAAGCGGATCAGGATCTGGAGTAGCCTCAGGTACTACAGGTACTGGCAACGAGTCAAACTCTGAACTGGCTACAAATGCAAGTCAACAACCCAACTCTGGAGTGGGTAGTGGAGCTGGTTCCGGTTTGAGTGGCAGTGAAGCAGGTGGTACGTCACCTGGAGCTGCTAGTACTGGAACTGACCAAGAGTCAGGTGTTGGCCCCGGTCAAGCGTCAAGCGTTGCATCTGATTCTACCTCAGCTGTTTCAAACACCCCACAATTTTCACCACTTGCCGGAGGCGCTGCTGCTAAAGGTTACACAATTGGAAGTATGGTGATAGCATTTGCAATGATCC**T**ACTCTAGATCTAAGTTACTTTAAAACAATCTTTCAACAACTTTGTCGACGATTTGTATTTCGGTTATGTTTACAAGTTTTATGA

**>Repaired *CORT_0E05950* sequence**

ATGTACCCAAGCATTTACGAGAGATTGGATCAAAGGGAGGCCTATAACAAACAAAAAATATAAAAGGAGCCATAAATTACAACTTTTCAACCATTGCAATAAAATTATCATTAAGTTTGTCAATCGCTTTAACATC

ATGAGACTTTCAACAATCCAGTTGTTAGTCTATTTTGTGTGCTCAATACAAGCCTCCCTGGTTTTCTTTCCAAATTCATTCAATGATTGCACCCCACCAACAAAACCTGTCTGCACGGTAAATGAAGTTTACTGCGACAACAACTCCAACAATCCACAATATAGTAACGCCAATTTTAATGCCGCTTTTGTTGTGTCTGAAGCTGAAAAGAATGCTGATGGTAGTTATAATGTTGTTGGTAATTATCAAGCTGCTCAATCAGACCAATTGTTTAACAATTTTGCACAGAACGTTGATCAAATCTACATTAGCGGAACAGGTGTCAATGATGCAACTATTTACAGTCAATCAACCAACAACCCAGTCAACAATCCTTTCAAATGGTCAGCCAAATTTCGCTGTCAACCGCAGATTAAATTTGGCAAGTGCTGTATTCCGGATGGTTTGAAACTCTACTATAAATTTAAACAAGGTGGTGCAGGGGGTGCAGTGTTGAATACGGTTTTTGGACAACAACAACTAAGCTACTACAATACCAACCCTTTATCTGGGGACGCTCAACTATATGATCCCAACAATTTGTTCAATCAACAATTGTCGTTTCACAAGAGAGACGAAGAAGCTGATGTCGACCAGAGAACTGAATTGAGTAAAAGGACCTCAAATGGGTTGCTCAATTTATTGATTAGCTGCACTAATAAGCACCAAGGAATTAAGCAATTCTGTTGGGATTGTG**ATTGTACTCCACCTTCTAGCTCTTCAGAACCGCCATCAAGTTCAGAGCCACCAAGTTCTTCTTCAGAGCCACCTAGCTCTTCAGAACCGCCATCAAGTTCAGAGCCACCTAGCTCTTCAGAACCCCCATCAAGTTCAGAGCCACCTAGTTCTTCTTCAGAGTCACCAAGCTCTTCAGAACCACCATCAAGTTCAGAGCCACCAAGTTCTTCTTCAGAGTCACCAAGCTCTTCAGAACCACCATCAAGTTCAGAGCCACCTAGCTCTTCAGAACCGCCATCAAGTTCAGAGCCACCTAGTTCTTCTTCAGAGCCACCTAGCTCTTCAGAACCGCCATCAAGTTCAGAGCCACCTAGTTCTTCTTCAGAGCCACCTAGCTCTTCAGAACCGCCATCAAGTTCAGAGCCACCTAGTTCTTCTTCAGAGCCACCTAGCTCTTCAGAACCGCCATCAAGTTCAGAGCCACC**A**AGTTCTTCTTCAGAGCCACC**A**AGCTC**A**TC**G**TCAGAGCCA**C**C**T**AG**C**TCAGAGCCACCTAGTTCTTCTTCTTCAGAACCACCATCAAGTTCAGAGCCACCTAGCTCTTCAGAACCACCATCAAGTTCAGAGCCACCTAGCTCTTCAGAACCACCATCAAGTTCAGAGCCACCTAGCTCTTCAGAACCACCATCAAGTTCAGAGCCACCTAGCTCTTCAGAACCACCATCAAGTTCAGAGCCACCTAGCTCTTCAGAACCACCATCAAGTTCAGAGCCACCTAGCTCTTCAGAACCACCATCAAGTTCAGAGCCACCTAGTTCTTCAGAACCGCCATCAAGTTCAGAGCCACCAAGCTCATCATCAGAGCCACCAAACTCATCGTCAGAGCCACCAAACTCATCGTCAGAACCACCAAGCTCATCATCTTCTCCACCTAGTACTACTACTCCACCATTCACTGCAGTGATTGAAACATCAACCATTGATTCTACAACAGTGATCACCATCACTTCTTGCTTTTATGGAGGCTGCTCTACAATCACTGAAACTACTGGATTGACGGTTATTACCGAAGGAACAACTGTTTACACTACTTATTGTCCCTTAACAGGAGCAACAATTACTTCAACTACCACTGGTAAGGGACCAAGTAATGGATCAGGAAGCAACAATGGGTCCGGTCATGGAAGTGGATCAGGAAGCGGATCAGGAAGTGGATCAGGAAGCGGATCAGGAAGTGGATCAGGAAGTGGATCAGAAGGTAATAATGGGTCGGGTAATGGGTCGGGTAATGGATCAGGAAGTGGGTCAGGAAGCGGATCTGGAAGTGGATCAGGAAGTGAATCTGGAAGTGGTGCTGTTTCAAGTAACAACAATGGATCAGGTTCTACCACTGTTATTACAGTCACGACTTGCTCAAAGGGTGGTTGCTCCACTTTGACTGAAACTACCGGTGTCACGGTAATTACCGAAGGGACAACCATTTACACCACTTATTGTCCTTTGACTGGTGAGGCACCGCCTTCATCTTTATCTATAGCTCCAAGTAATGTTTCTGGTTCTGGAAGC**GGATCAGGAAGTGGATCAGGAAGTGGATCA**GG**AAG**CGG**AT**C**A**G**G**ATCTAGCCATGAAAGCGGATTAGAAGGTAAC**AATGGGTCGGGTAATGGATCAGGAAGTGG**A**ACAGGAAGCGGATCAGGATCTGGAGTAGCCTCAGGTACTACAGGTACTGGCAACGAGTCAAACTCTGAACTGGCTACAAATGCAAGTCAACAACCCAACTCTGGAGTGGGTAGTGGAGCTGGTTCCGGTTTGAGTGGCAGTGAAGCAGGTGGTACGTCACCTGGAGCTGCTAGTACTGGAACTGACCAAGAGTCAGGTGTTGGCCCCGGTCAAGCGTCAAGCGTTGCATCTGATTCTACCTCAGCTGTTTCAAACACCCCACAATTTTCACCACTTGCCGGAGGCGCTGCTGCTAAAGGTTACACAATTGGAAGTATGGTGATAGCATTTGCAATGATCC**TG**ACTCTAGATCTAAGTTACTTTAAAACAATCTTTCAACAACTTTGTCGACGATTTGTATTTCGGTTATGTTTACAAGTTTTATGATTTCGGTATTTTCTATTTGATTTCAGTTTAGCTTCATTTAGAGAGTATATATAATTTACTTCAATATATTCGTAATTCTTTTAAATGTTTCTCTATTTTTTCTTTGTTTTGCCTTCTTATCTCTCAACAGTCAT

The original CORT_0E05950 translation predicted a much-shorter protein that was annotated as Cdc24 GDP-GTP exchange factor (below). The protein predicted from the corrected *CORT_0E05950* has a putative signal peptide processing site (bold type). The predicted protein does not have a predicted GPI anchor addition site with the best potential site bolded and underlined below. CORT_0E05950 was a “hit” from blastp searches using *C. albicans* Rbt1 as the query. A sequence alignment between CORT_0E05950 and *C. albicans* Rbt1, created using Clustal Omega (<https://www.ebi.ac.uk/Tools/msa/clustalo>), is also shown.

**>Original CORT_0E05950 translation**

MRLSTIQLLVYFVCSIQASSVFFPNSFNDCTPPTKPVCTVNEVYCDNNSNNPQYSNANFNAAFVVSEAEKNADGSYNVVGNYQAAQSDQLFNNFAQNVDQIYISGTGVNDATIYSQSTNNPVNNPFKWSAKFRCQPQIKFGKCCIPDGLKLYYKFKQGGAGGAVLNTVFGQQQLSYYNTNPLSGDAQLYDPNNLFNQQLSFHKRDEEADVDQRTELSKRTSNGLLNLLISCTNKHQGIKQFCWDC

**>CORT_0E05950 = Predicted protein from repaired *C. orthopsilosis* ORF**

**MRLSTIQLLVYFVCSIQA**SLVFFPNSFNDCTPPTKPVCTVNEVYCDNNSNNPQYSNANFNAAFVVSEAEKNADGSYNVVGNYQAAQSDQLFNNFAQNVDQIYISGTGVNDATIYSQSTNNPVNNPFKWSAKFRCQPQIKFGKCCIPDGLKLYYKFKQGGAGGAVLNTVFGQQQLSYYNTNPLSGDAQLYDPNNLFNQQLSFHKRDEEADVDQRTELSKRTSNGLLNLLISCTNKHQGIKQFCWDCDCTPPSSSSEPPSSSEPPSSSSEPPSSSEPPSSSEPPSSSEPPSSSEPPSSSSESPSSSEPPSSSEPPSSSSESPSSSEPPSSSEPPSSSEPPSSSEPPSSSSEPPSSSEPPSSSEPPSSSSEPPSSSEPPSSSEPPSSSSEPPSSSEPPSSSEPPSSSSEPPSSSSEPPSSEPPSSSSSEPPSSSEPPSSSEPPSSSEPPSSSEPPSSSEPPSSSEPPSSSEPPSSSEPPSSSEPPSSSEPPSSSEPPSSSEPPSSSEPPSSSEPPSSSEPPSSSSEPPNSSSEPPNSSSEPPSSSSSPPSTTTPPFTAVIETSTIDSTTVITITSCFYGGCSTITETTGLTVITEGTTVYTTYCPLTGATITSTTTGKGPSNGSGSNNGSGHGSGSGSGSGSGSGSGSGSGSGSGSEGNNGSGNGSGNGSGSGSGSGSGSGSGSESGSGAVSSNNNGSGSTTVITVTTCSKGGCSTLTETTGVTVITEGTTIYTTYCPLTGEAPPSSLSIAPSNVSGSGSGSGSGSGSGSGSGSGSSHESGLEGNNGSGNGSGSGTGSGSGSGVASGTTGTGNESNSELATNASQQPNSGVGSGAGSGLSGSEAGGTSPGAASTGTDQESGVGPGQASSVASDSTSAVSNTPQFSPLAGGAAAKGYT**I**GSMVIAFAMILTLDLSYFKTIFQQLCRRFVFRLCLQVL

**>C4_03520C_A = orf19.1327 = *C. albicans* Rbt1**

**MRFATAQLAALAYYILSTEA**TFPLLGDIFNCIPHNTPPVCTDLGLYHDSSISLGGSKNKREAEIANKDGTIEKRTFGSAGVNAGFNAAFVVSNAKKLSDGSYGIDCNFKSDSSVQLNSAFGKKVKQLSITGTGYSDISLLGNVANPFEWSASLKVKAEIVKGKCCLPSGFRIVTDFESNCPEFDAIKQFFGSSQIIYKVNAVSNAIGTFDASALFNAQVKAFPAKRELDEFEELSNDGVTHSKRTLGLLLGLLKKVTGGCDTLQQFCWDCQCDTPSPSTTTVSTSSAPSTSPESSAPSTTTVTTSSSPVTSPESSVPETTTVTTSSVPETTPESSAPETTTVTTSSVPSTTPESSAPETTPESSAPESSVPESSAPETTPESSAPESSVPESSAPETETETTPTAHLTTTTAQTTTVITVTSCSNNACSKTEVTTGVVVVTSEDTIYTTFCPLTETTPVPSSVDSTSVTSAPETTPESTAPESSAPESSAPESSAPVTETPTGPVSTVTEQSKTIVTITSCSNNACSESKVTTGVVVVTSEDTVYTTFCPLTETTPATESASESSAPATESVPATESAPVAPESSAPGTETAPATESAPATESSPVAPGTETTPATPGAESTPVTPVAPESSAPAVESSPVAPGVETTPVAPVAPSTTAKTSALVSTTEGTIPTTLESVPAIQPSANSSYTIASVSSFE**G**AGNNMRLTYGAAIIGLAAFLI

CORT_0E05950 MRLST--IQLLVYFVCSIQASLVFFPNSFNDCTPPTKPVCTVNEVYCDNNSN-------- 50

orf19.1327 MRFATAQLAALAYYILSTEATFPLLGDIFNCIPHNTPPVCTDLGLYHDSSISLGGSKNKR 60

**::* : *.*:: * :*:: :: : ** * **** :* *.. .

CORT_0E05950 ----------------NPQYSNANFNAAFVVSEAEKNADGSYNVVGNYQAAQSDQLFNNF 94

orf19.1327 EAEIANKDGTIEKRTFGSAGVNAGFNAAFVVSNAKKLSDGSYGIDCNFKSDSSVQLNSAF 120

. **.********:*:* :****.: *::: .* ** . *

CORT_0E05950 AQNVDQIYISGTGVNDATIYSQSTNNPVNNPFKWSAKFRCQPQIKFGKCCIPDGLKLYYK 154

orf19.1327 GKKVKQLSITGTGYSDISLLG-----NVANPFEWSASLKVKAEIVKGKCCLPSGFRIVTD 175

.::*.*: *:*** .* :: . * ***:***.:: : :* ****:*.*::: .

CORT_0E05950 FKQGGAGGAVLNTVFGQQQLSYYNTNPLSGDAQLYDPNNLFNQQLSFHKRDEEADV---- 210

orf19.1327 FESNCPEFDAIKQFFGSSQIIY-KVNAVSNAIGTFDASALFNAQVKAFPAKRELDEFEEL 234

*:.. .:: .**..*: * :.* :*. :* . *** *:. . ..* *

CORT_0E05950 -DQRTELSKRTSNGLLNLLISCTNKHQGIKQFCWDCDCTPPSSSSEPPSSSEPPSSSSEP 269

orf19.1327 SNDGVTHSKRTLGLLLGLLKKVTGGCDTLQQFCWDCQCDTPSPSTTTVSTSSAPSTSPES 294

:: . **** . **.** . *. : ::******:* ** *: *:*. **:* *

CORT_0E05950 PSSSEPPSSSEPPSSSEPPSSSEPPSSSSESPSSSEPPSSSEPPSSSSESPSSSEPPSSS 329

orf19.1327 ---SA------PSTT--------------------TVTTSSSP----VTSPESSVPE--- 318

* * :: :**.* **.** *

CORT_0E05950 EPPSSSEPPSSSEPPSSSSEPPSSSEPPSSSEPPSSSSEPPSSSEPPSSSEPPSSSSEPP 389

orf19.1327 ---------TTTV--------------TTSSVP-E---TTPES----------------- 334

::: :** * . *.*

CORT_0E05950 SSSEPPSSSEPPSSSSEPPSSSSEPPSSEPPSSSSSEPPSSSEPPSSSEPPSSSEPPSSS 449

orf19.1327 --SAP------ETTTV----TTSSVPSTTPESSAPETTPES------------------- 363

* * ::: ::*. **: * **: . *.*

CORT_0E05950 EPPSSSEPPSSSEPPSSSEPPSSSEPPSSSEPPSSSEPPSSSEPPSSSEPPSSSEPPSSS 509

orf19.1327 -----SA------P-ESS------VP-ES------SAPET---------TPESSA----- 384

* * .** * .* * * : *.**

CORT_0E05950 EPPSSSEPPSSSSEPPNSSSEPPNSSSEPPSSSSSPPSTTTPPFTAVIETSTIDSTTVIT 569

orf19.1327 --PESSVPE-----------------------SSAPETETETTPTAHLTTTTAQTTTVIT 419

*.** * **:* : * ** : *:* ::*****

CORT_0E05950 ITSCFYGGCSTITETTGLTVITEGTTVYTTYCPLTGATITSTTTGKGPSNGSGSNNGSGH 629

orf19.1327 VTSCSNNACSKTEVTTGVVVVTSEDTIYTTFCPLTETTPVPSSVDSTSVTSAPETTPEST 479

:*** ..**. ***:.*:*. *:***:**** :* . ::... ..: ... ..

CORT_0E05950 GSGSGSGSGSGSGSGSGSGSGSGSEGNNGSGNGSGNGSGSGSGSGSGSGSGSESGSGAVS 689

orf19.1327 AP-----------------ESSAPE----------------SSAPESSAPVTETPTGPVS 506

. ..*. * *.: ..*. :*: :* **

CORT_0E05950 SNNNGSGSTTVITVTTCSKGGCSTLTETTGVTVITEGTTIYTTYCPLTGEAPPSSLSIAP 749

orf19.1327 --TVTEQSKTIVTITSCSNNACSESKVTTGVVVVTSEDTVYTTFCPLTETTPATESASES 564

. . *.*::*:*:**:..** . ****.*:*. *:***:**** :* :. :

CORT_0E05950 SNVSG-----SGSGSGSGSGSGSGSGSGSSHESGLEGNNGSGNGSGSGTGSGSGSGVASG 804

orf19.1327 SAPATESVPATESAPVAPESSAPGTETAPATESAP---------------ATESSPVAPG 609

* : : *. : ..*. *: :. : **. : ..* ** *

CORT_0E05950 TTGTGNESNSELATNASQQPNSGVGSGAGSGLSGSEAGGTSPGAASTGTDQESGVGPGQ- 863

orf19.1327 TETTPATPGAESTPVTPVAPESS--------APAVESSPVAPGVETTP---VAPVAPSTT 658

* * .:* : : *:*. . *:. .:**. :* : *.*.

CORT_0E05950 --ASSVASDSTS----AVSNTPQF-------------SPLAGGAAAKGYTIGSMVIAFAM 904

orf19.1327 AKTSALVSTTEGTIPTTLESVPAIQPSANSSYTIASVSSFEGAGNNMRLTYGAAIIGLAA 718

:*::.* : . ::...* : * : *.. * *: :*.:*

CORT_0E05950 ILTLDLSYFKTIFQQLCRRFVFRLCLQVL 933

orf19.1327 FLI-------------------------- 721

:*
