# Supplementary material for: Development and Use of a Monoclonal Antibody Specific for the Candida albicans Cell-Surface Protein Hwp1
Source: Front Cell Infect Microbiol. 2022 Jun 27;12:907453. doi: 10.3389/fcimb.2022.907453 (PMC9273023; doi:10.3389/fcimb.2022.907453)
Supplement: Supplementary file 4 [file Table_1.docx]

**SUPPLEMENTARY TABLE S1.** Amino acid sequences corresponding to the entries in **Table 1**.

| **CGOB Pillar** | **Sequence Identifiers** | **Protein Sequence*** |
| --- | --- | --- |
| *Candida albicans* Hwp1 | *C. albicans* Hwp1; C4_03570W_A; orf19.1321 | **MRLSTAQLIAIAYYMLSIGATVPQVDG**QGETEEALIQKRSYDYYQEPCDDYPQQQQQQEPCDYPQQQQQEEPCDYPQQQPQEPCDYPQQPQEPCDYPQQPQEPCDYPQQPQEPCDNPPQPDVPCDNPPQPDVPCDNPPQPDVPCDNPPQPDVPCDNPPQPDQPDDNPPIPNIPTDWIPNIPTDWIPDIPEKPTTPATTPNIPATTTTSESSSSSSSSSSSTTPKTSASTTPESSVPATTPNTSVPTTSSESTTPATSPESSVPVTSGSSILATTSESSSAPATTPNTSVPTTTTEAKSSSTPLTTTTEHDTTVVTVTSCSNSVCTESEVTTGVIVITSKDTIYTTYCPLTETTPVSTAPATETPTGTVSTSTEQSTTVITVTSCSESSCTESEVTTGVVVVTSEETVYTTFCPLTENTPGTDSTPEASIPPMETIPAGSEPSMPAGETSPAVPKSDVPATESAPVPEMTPAGSQPSIPAGETSPAVPKSDVSATESAPAPEMTPAGTETKPAAPKSSAPATEPSPVAPGTESAPAGPGASSSPKSSVLASETSPIAPGAETAPAGSSGAITIPESSAVVSTTEGAIPTTLESVPLMQPSANYSSVAPISTFE**G**AGNNMRLTFGAAIIGIAAFLI |
|  | *C. dubliniensis* Hwp1; Cd36_43360 | **MKLSTAKLVAIAYYMLSIGATIPSVDD**QQVEEGLIQKRTYGYYQEPCDDYYPPQQQQEEPCDYPQQQPQEPCDNPPQPEEPCDNPPIPNIPTDWIPNIPTDWIPNIPNEPTNPPTTPNIPATTTTSESSSVPSTTPKTSASTTPESSVSATSPYTSVPTTIPKSSTPATGPETSVPGSSILATTSESSSVRATTPNTSVPTTASESSTLETKTSITPLTTSTEHDTTVITVTSCSNSACAESEVTTGVVVVTSEDTIYTTYCPLTETTPGTESTPEASTPIMETIPADSEPSVPASETSAVVPELSVPTTESAPGTKMTPGGLEPSAPVSEMTPAGIKTNQAVPESSIPAGSSVAITTPVNSTIVSTTEGAIPTTLESVPIMQPSANYSSSVAPVSTFE**G**VGNNIRLTYGAAIVGLAALLI |
|  | *C. tropicalis* CTRG_00478; (BLAST suggested this protein was more like Hwp2 than Hwp1 but it was placed into the *HWP1* pillar on CGOB) | **MRFTTTQLVAIAFYLASIEA**TFSLGTIFDYVPKDDAPVCKQLRIYTDESTNSGHKRDDIQNDDDLEKRYFSKGISIGADVSAEKNIGLGISKGASVDAGISKNIGAGVSAGKNVAVDVSKNIGVGVGFGVGAAVGVGAAIGAEVSKSVGVGAGATDVAEVSKSVGAGVTIGAEVSKSIGAGINVDKTIDAGFSKTISVGVSAEKTAGVDISKHIGAGISIGKTIGADISKNIQAGVAAGLSISKGADVEVSKNIGAGVSIGKNLGVGISKGIDASAGVGVSAEKNVGLGISKNIGVDISADKSVSVGKRNINLGVNLGAAVGASVGAGVSAEKNLGVSIGAGASAGASSSVGVNAAFVVSSVSKLSDGNYGVTCNFKCDSSAKLNSAFAKKIKNLSIVGTGCGDVSIYGEGAIEKVSNPFEWSTSIKVKPEIINGKCCLPSGFQIVADFEENCDELDAFKEIFGKEKFIYKLNSVISAAKTFDASVLFNAQASAFVEKRSIQETRPYTNNTIIHDKRSLSLLLSLLKKVSVGGGCDSLPQFCWDCDCETTTSTTSTEECTESQGTSSTSASSCTPSTTSITSTDSTTSTSTTSTSTTPTSTTSTSTTLTTSTSTASTDSTTFTKSSVQLSSKSSAESSTEHAASNSEENISSKFESSVQSSTSVELSTESSTESSTELSTESSTESSTESSTESSTESSTESSTESSTESSTESSTESSTESSIQSSVESSESSTDFASTSDPESFVQYSVISSESSESSIKHMSTTDNSQFLSTQTEHSTTILTVTSCSNDVCTETAITTGVTVVTEDETIYTTYCPLTDESTPVEESTSANTSSLLASTTDKEFIHQHDKSSLVSNGNALSTETEKLTTVITVTSCSNDACSETPITTGVRVVTSDETTYTTYCPLSSYSDIASVEENVSSVSTLIETPAAYFEGAECDTTSTVTDFKTIVIDTEGTVPTTVEFVSLIPVSSLAEPSTVVAITTFE**G**AANNMRLPVGAAILGLVAMMV |
|  | *C. parapsilosis* CPAR2_403520, CPAG_01519 | **MRLHIFQLLAFIVSICSA**DVFFPGSFKDNTLPLLPPVCTEDKLYCETTYNPTYFMTTFNAAFVVSESKRNIDSSFSIVINYQADKYDQLYNNFLQNIGQIHLTGTGTADPILYSYGIPNTITNPFRWSARIQVTTQVKNGKCCTRDGMRLWYTFRPYGTGYAALYQVFGTQQIYYTTNAWNGAVQQYDPMTLFNQQLLFQKREEEKCDANELAHLNKRDYSSQVSLLNSCTTKHYGIKQFCWDCGCPPPPPPPSSPPPPSSPPPSSPPPPPSSPPPSSEPPSSEPPSSEPPSSEPPSSEPPSSEPPSSSEPPSSSEPPSSEPPSSEPPSSEPPSSSEPPSSEPPSSEPPSSEPPSSSEPPSSEPPSSEPPSSSEPPSSEPPSSEPPSSEPPSSSEPPSSEPPSSNPPSSEPPSSNPPSSEPPSSNPPSSEPPSSEPPSSSEPPSSSEPPSSEPPSSNPPSLNPSSNPPSSNPPSSNPSSNPPSSNPPSSNPSSNPPSSNPPSSNPSSNPPSSNPPSSNPSSNPPSSNPPSSNPSSNPPSSNPSSNPPSSNPPSSNPSSNPPSSNPSSNPPSSNPPSSNFPSSGGSSGPLSSTTSPSSIVQSSSVNLSSSLSETTSKSSIASLESTESSGQESSNVISTTIVTITTCLHGSCSTNTETTGLTVITEGTTTYTTYCPLTEATATSTAGGGRGSGSGNGSGNGSGNGSGNGSGNGSGNGSGNGSGNGSGNGSGNGSGNGNGQPSVTTITITTCSNGGCSTILETTGVTVITRGTNVYTTYCPLTSQTSDSSPTKGPINGSGSGSGSGPGSGPGSGSGSGSGSGSGSGSGSGSGSGSQSSSLTIVTVTTCLNGDCSIVVETTGVTVVTEGTTTYTTYCPLPAEMTLSSTSIGPSSESGNGNKPGNEGAGPANQGLHPASGEQGSGSGSPEGQGSSPGSPGSPEGQGSGSGSPEGQGSSPGSPEGQGSSPGSPEGQGSSPGSPEGQGSSPGSPEGQGSSPGSPEGQGSSPGSPEGQGSSPGSPEGQGSSPGSPEGQGSSPGSPEGQGSSPGSPEGQGSSPGSPEGQGSSPGSPEGQGSSPGSPEGQGSSPEGQGGQGSSPGSPEGQGSSPGSPEGQGSSPGSPEGQGSSPEGQGGQGSSPGSPEGQGSGSGSPEGQGSSPGSQMSIVQESSQGSIVVSPTVLHSVEGLIPSLETVSIMQSSESSSSESNPAEISAFA**A**VGATIGYSIGAISLAFAVILL |
|  | *C. orthopsilosis* CORT_0E03570  (No signal peptide) | MTLFNQQLMFQKRDLEDKGDAGELDTLDKRSYQSMVELLNSCTTKHHGIKQFCWDCGCAPPPPPPSSPPPSSEPPSSSEPPSSEPPNSEPPSSEPPSSSEPPSSRPSPPSSESSSSSKPSSSIVQPSSESFSSSMPETTSRSSSTSLESTESSGLSISGISGLSTARSTTIITVTTCAHGSCSIITETTGVTIITEGTKIFTTYCPLTGESSPSSSTIGPSNGSGGKGGGNGSNNVGGNGNGSGSGNGNGSRSSTVIAVTTCSNGGCSTVVETTGVTVRTEGTTIYTTYCPLTGETIPSSLTSVPSNGSGAGSGTGQGAGQTAGQGSGSGPSQGSGPGAGQESIETSLTEQQSTENHIPSLQTVSIMQSSESSSSESNPAEISVLA**A**VGPTIGYSIGAVFLAITMILL |
|  | *C. metapsilosis*  Cm_5_551924, CMET_360 | **MILLFAQLFALFTCVKA**SLVFFPNSFKDNTLPLQPALCDVEKLYVDSQQPATFFNPTFNAAFVVAQSERMWDGSYKIVVNYQADKYDQLYNNFLQNIAQVYFTGTGVTDSTVYITGIPNSITNPFQWSAKIQVRPQLKNGKCCIMDGFRLWFRFNQLGTGGAALNQVFGQQNSWYNTQVLSGPFFQYDPMTYFNQQLVFRKRDAEQCDANEVEGLDKRSYQSMVDLLNSCTTKHRGIKQFCWDCNCPPPPPPPSSPPPSSPPPSSPPPSSPPPSSPPPSSPPPSSPPPSSVPPSSEPPSSEPPSSPPPSSELPSSELPSSEPSSELPSSELPSSEPSSEPSSEPSSEPSSELPSSEPSSELPSSEPSSEPSSELPSSEPNSEPSSEPPSSELPSSEPSSEPSSEPPSSELPSSEPSSEPSSEPPSSELPSSEPPSSELPSSEPSSEPSSEPSSELPSSEPSSELPSSEPSSEPSSEPPSSELPSSEPSSEPSSEPPSSELPSSEPPSSELPSSEPSSEPSSEPSSELPSSEPSSEPPSSVPSSEPPSSEPPSLSEPPSSSEPPISEPSSELPSSGPSSTEPSSEPSSEPSSEPSSGPPSSEPPSSGPSSEPPSSSEPPSSEPPSSSEPPSSEPSSGPPSSEPPNSEPSSGPPNSEPSSGPPSSGPPSSGPSSEPPSSSEPPSSEPPSSSEPPSSEPSSGPPSSEPPNSEPSSGPPSSGPSSGPPSSGPSSGPPSSGPSSEPPSSSEPPSSGPPSSEPSSGPPSSGPPSSEPSSGPPSSGPSSEPPSSEPSSGPPSSEPSSEPSSGSPSSQPSSGPSSGPSSEPPSSFGPPSSQPPSSSEPSSSSEPTASSGPQSLSSETSISSSTISSPRPVSSSPSFTQVIETSTVESTTVITTTTCSHGVCSTITITTGLTVITEGTTVYTTYCPLTGATTTSTTAGGSGAGNSNGNGSGSGSGSGSANGSGNGNGSGSGSGSGSGSGNGNENGSGPGIGNGSGSGSTTVITTTACRNGGCSIITKTTGVTVITEGTTTYTTYCPLSGATTPPTATEGAGSGNGNNNGNGQESGHGNGENNGNGSGSDASSSNGSGSGSANGPGAGSGSGAGSGSGAGSGSGAGSGSGAGSGSGAGSGSGAGSGSGAGSGGGQGTGSDTGNGSENGSGGSGASPGASPGTSPGTSPGASPGASPGSGQGASPGASPGSGQGASPGASPGASPGSGQGASPGASPGTSPGASPGTSPGASPGASPGLGQGPSPGASPGASPGSSPGSSPGSGEESGFGEGTGSESQGANQESLKASPTEQHLSENLMPSFETVSIMQSSESSPSESNPVEISAFAAI**G**ATIGYSFGVVSFAIAIILL |
| *Candida albicans* Rbt1 | *C. albicans* Rbt1  C4_03520C_A  orf19.1327 | **MRFATAQLAALAYYILSTEA**TFPLLGDIFNCIPHNTPPVCTDLGLYHDSSISLGGSKNKREAEIANKDGTIEKRTFGSAGVNAGFNAAFVVSNAKKLSDGSYGIDCNFKSDSSVQLNSAFGKKVKQLSITGTGYSDISLLGNVANPFEWSASLKVKAEIVKGKCCLPSGFRIVTDFESNCPEFDAIKQFFGSSQIIYKVNAVSNAIGTFDASALFNAQVKAFPAKRELDEFEELSNDGVTHSKRTLGLLLGLLKKVTGGCDTLQQFCWDCQCDTPSPSTTTVSTSSAPSTSPESSAPSTTTVTTSSSPVTSPESSVPETTTVTTSSVPETTPESSAPETTTVTTSSVPSTTPESSAPETTPESSAPESSVPESSAPETTPESSAPESSVPESSAPETETETTPTAHLTTTTAQTTTVITVTSCSNNACSKTEVTTGVVVVTSEDTIYTTFCPLTETTPVPSSVDSTSVTSAPETTPESTAPESSAPESSAPESSAPVTETPTGPVSTVTEQSKTIVTITSCSNNACSESKVTTGVVVVTSEDTVYTTFCPLTETTPATESASESSAPATESVPATESAPVAPESSAPGTETAPATESAPATESSPVAPGTETTPATPGAESTPVTPVAPESSAPAVESSPVAPGVETTPVAPVAPSTTAKTSALVSTTEGTIPTTLESVPAIQPSANSSYTIASVSSFE**G**AGNNMRLTYGAAIIGLAAFLI |
|  | *C. dubliniensis* Rbt1  Cd36_43400 | **MRFATAQLAALAYYILSTEA**TFPLLGDIFDCIPHNTPPVCSELNLYHDTSASINGSQNNKNKRDVEKRTFGNFGFNSGVNAAFVVSGAKKLSDGSYGIDCNFKSDSSVQLNSAFGKKIKQLSVTGTGYSDIDLLGHVANPFEWSASFKIKAEIIKGKCCLPSGFKIITDFESNCPEFDAIKQIFGTSQIIYKVNAISHAIGTFDASALFNAQLNAFPVKRELNEFEELNNNNGITHSKRTLGLLLNLLKKVTGGCDTLQQFCWDCQCDTPSVSTTIVSTSSAPETSTTPESSVPETTIVTTSSAPETTPESSAPETTTTTTPESSAPETTTTTTPESSAPETTPVSSSVDSTSVTSVPESSAPETTPVSSNVDSTSVTSVPESSAPESSAPETETSPIVQLSTTTAQTTTVITVTSCSNNACSKTKVTTGVVVVTSEDTIYTTFCPLTETTPVTSNVDSTAETSAPESSVPETTAESTTPESTAPESTVPETTAESTTPESTAPESTAESTTPESTAPETETSPTVQFSTTTAQTTTVLTVTSCSNNACSESEVTTGVVVITTEDTIYTTFCPLTETTTATESSTPSVETTPIAPESSAPASESVPVTESSSVAPIAETTPVSPVAPNAESTPVAPNAESTPVAETTPIAPTTAPETSAIVSTTEGTIPTTLESISIIQSSANSSYTIAPVSSFE**G**AGNNMRLTYGAAIVGLAALLI |
|  | *C. tropicalis*  CTRG_00477 | **MRFATTQLVSLAFYLVSIEATYA**ISGLFDCIPKTEVPVCPTLDLYTDTSVSVGSKREDQFEDDLEKRTFLNAGLNLGASVGSSAGINAAFVVTGAKKLPDGNYGITCNFKSHSAKNLKSSFASKIQNLKIVGTGCGDVPLYGKGCTKYVANPFDWSASFKVKPQVINGKCCLPADFQIVTDFKSNCLQLDAIKQIFGGDKLIYKLNTIGSAMETFDSNLLFKSQCDFFGIKRDLENIEQYDNVGITHSKRTLGVFLDLLKSVSGGGGCNTINQFCWDCDCDTTTPTTSSTAVCTECEGTTSTGTSSILSSSSTGQSTTCTTKKTTPVTTEETTPVTTEETTPVIYEETTSATTEETTPATTEETTPATTKETTPVTTEETTPVTTKETTPATTEETEETTPATTEETTPATTEETTKETTKETTPATTEETEEITPATTEETEETTKETTPATTEETEETTPATTEETTKETTKETTKETTKETTEETEETTPATTEETEQSSSIASTTAPAPEESSPAEETPEESSPAEESPEESSPAEESPEESSPAEESPEESSPAEETPEESSQSEETTVPLSTQTEHSTTILTVTSCSSDVCTETSITTGVTVVTSAETIYTTYCPLTEESTSSTDKSPEETTPATTEETEQSSSIASTTAPAPEESSPAEETPEESSPAEESPEESSPAEETTVPLSTQTEHSTTILTVTSCSNDVCTETSITTGVTVVTSAETIYTTYCPLTEESTTSVEETSEETTSAKSSSVASTTAPAPSEEETTSSAQGEEETTPAAPAPEETTTSKESSSIASTTAPAPEETTPAAPAPEETTPAAPAPEETTPAAPAPEETTPATTCEGEGCEVFVISTSTTIGTSTVVPVTEGHIPTTVESVSVTPVASPAEPSSIDLITNFE**G**AGNNMRLSFGAAIVGLAAFLI |
|  | *C. parapsilosis*  CPAR2_403510, CPAG_00831 | **MKLSTASLVSLAALVGSSEA**TWALFEDLFKDCAPKHVKPICKIKDIFCDTDDSNESSVSARDGGSVNFKAAFVVSGAERNDDGTYNVVANYEADQSDQLHQYFGGDIDSLSLTGTGCDDVHLYGKSASDSVENAFKWSTKFQCKPKIKNGKCCLPDGFSVGYDFSESGSSWDALKSVFGSKSCSYGINTQWSFVKSFDPYALLNKELSLFKRDEVVEGVDGSTTFDKRTLGFLLDLLGKCTTKKKIIKQFCWDCECPSTSSSSTVPPSTSSTPPTESSTPPTESSTPPTESSTPPTESSTPPTESSTPPTESSTPPTESSTPPTESSTPPTESSTPPTESSTPPTESSTPPTESSTPPTESSTPPTESSTPPTESSTPPTESSTPPTESSTPPTESSTPPTESSTPPTESSTTPTESSGKPSESSTTPTESSGKPSESSTTPTESSVKPSESSTTPTESSGKPSESSTTPTESSVKPSESSGKTTTESSTESSGKPSESSTESSGKPSESSTPSGPTDTVTTHSTTVVTITSCSEDKCETSTATTGVTVITEGTTIYTTYCPLTEEETTSTVPTSKQTPIPETTSTTAETTVPTKGGESETENVPSTLTTTTAAPSSSAPTGEGEASKSEGESQTTAAPPASEGEAPKSEGESQTTVAPTGEAPKSEGESQTTAAPTGEAPKSEGESQTTAAPTGEAPKSEGESQTIAAPSSAAPEGEAPKPEGESQTTAAPTGEAPKSEGESQTTAAPTGEAPKSEGESQTVAAESSSVPEVSQVPSESANETTAAVSTYE**G**AGARNSVGLLLVGAAALLL |
|  | *C. orthopsilosis*  CORT_0E03560 | **MKLSTANLLSLAAFVSSTQA**TWSLFEDLFKDCAPKHVQPICEIQDIFCDTDDSNDSVSARDASVNFKAAFVVSGAEKNDDGTYNVVANYEADQSDQLHQYFGGNIDSLSLTGTGCDDVQLYGQGASNAVSSAFKWSTKFQCKPEYKNGKCCLPDGFTIGFKFIEIGLAWEALKLVFGHQSCSYGIITHWSFLEVFDPSALLHHDLSLFKRDEVADGVEGFTTFDKRTLGFLHDLLGKCTTQNKGIKQFCWECDCPSTSSSTTVPPSTSSTPPTESSTPPTESSTPPTESCTPPTESSTPPTESSTPPTESSTPPTESSTPPTESSTPPTESSTPPTESSTPPTESSTPPTESSTPPTESSTPPTESSTPPTESSTPPTESSTPPTESSTPPTESSTPPTESSTPPTESSTPPTESSTPPTESSTPPTESSTPPTESSTPPTESSTPPTESSTPPTESSTPPTESSTPPTESSTPPTESSTPPTESSTPPTESSTPPTESSTPPTESSTPPTESSTPPTESYTSSGSTETVVTHSTTIVTITSCSEDKCETTTATTGITVITEGTTIYTTYCPLTEKTTSSATTSKETSVPETVSTITTASTGPTESNESGPESSTRVPTTTTATAPSTSVPVSSQPPSKSQSEAPKSEGESETAAAPTSESEAPKSEGESETAAAPSSSAPAGQSEAPSSAPGESQTVVSPSSAPASSQAPSEDQGQAPASEGESQTVEAQSSSTPKVSQAPSGGENETTAAISTYE**G**AAMRNSAGILLVGAAALLL |
|  | *C. metapsilosis*  Cm_5_537075  CMET_367 | **MKLTTTNLLSLAVLVCSTQATWC**LFEELFKDFAPKHVNPICEIKDILCDTDNSSDSYSAGADDSVNFKAAFVVSGAEKNDDGTYNVIANYEADQSDQLHQYFGSNIDLLSLTGTGVDDVQLYGNGASNAVSSAFKWSTKFQCKPEYKNGKCCLPDGFTVAYKFVEIGLAWDALKLVFGHKYCLYNVVTQWSFLKVFDPSSLLNNELSLFKRDEVADGIDGFSTIDKRTLGFLKDLLGKCTTKHKGFKQFCWECDCPSTSSSSTAPPSTSSTPATESSTPGTESSTPATESSTPATESSTPGNGSSTPATESSTPATESSTPATESSTPATESSTPATESSTPATESSTPATESSTPATESSTPATESSTPATESSTPATESSTPATESSTPGNGSSTPATESSTPGNGSSTPATESSTPATESSTPGNGSSTPTTESSTPGNGSSTPATESSTPSNGSSTPATESSTPGNGSSTPATESSTPATESSTPSNGSSTPATESSTPGNGSSTPATESSTPGNGSSSAQATESSSASSGSSTGSGLVLTATDHSTTVVTITSCSEDKCETSTATTGVTVVTEGTTIYTTYCPLTEESTSSTPASKTVPESGSTTTTSAGPSKGNESGSAPSSSTPAGQAAPSSAPAGQAAPSSAPAGQAAPGGQAAPGGQAAPSSSAPAGQAAPGGQAAPSSSAPASQGQAAPSSSAPASQGQAAPSSAPGGQGPAASGESQTVAAQSSSSSTPQVSQAPSQGGNENTAAVSTYE**G**AGVKNSVGALLIGAAALLL |
|  | *L. elongisporus*  LELG_04495 | **MKLSLASLTAIAVLLSSTDA**TFCLLDDSLFKDVVPKTVKPQCTPDQLYYDPSSSGVSKRTYGTSNFNAAFVVSNAQKNSDGTYNVVGNFQCDQADQLNQYFAKSVKGLSLQGTGCKDIKLFGADCDKPVENPFKWAANFQVQAEIVKGKCCIPKSLQIVTNFDEGCSEWSSLKQKFGSASISYKVSTIVNGIKTFDASSVFNSQLSLFKREEEADVLDDISGTSTYDKRTLGLLSDLLDKVTKGSKNCEIKQFCWDCECPGTSTESTGSSTVPPTTSCPPPVSSSIPPVSSSVPPVSSSVPPVSSSVPPVSSSAPPVSSSAPPVSSSVPPVSSSIPPVSSSVPPVSSSIPPVSSSVPPVSSSVPPVSSSVPPVSSSVPPVSSSVPPVSSSVPPVSSSVPPVSSSTPVKSTPVESSPVKSTPVESSPVESSPVESSPVKSTPVESSPVESSPVESSPVESSPVESSPVESTPVESSPVESTPVESTPVESSPVKSTPVESSPVKSTPVESSPVKSTPVESSPVKSTPVESSPVESSPVESTPVESSPVESSIHIDPPVESSPVESTPVESSPVESTPVESSPVESSPVESSTAPGESISTSLEGTASTLSSVPPKTVITHSTTIVTVTSCSENACSEQTHVTGVTVVTEDTTVYTTYCPLTEEVTASTEKTQPSEEAPSSTSPGAPESEASSESAKTSPGAPESEASSESAKTSPETTPIVKTVTAHSTTEITLTSCANDVCSEVTTSTGVAVVTEGTTVYTTYCPLTEEATITVQPSKEANEESAPGSAVETTSTAIVPPAATTEGSAPAPASASSSSSAEGPKGTATTTPAEWSEETVAAASSEAPKKTATAEKPESEETTVAAPSKGSEETAAASVAPPSKGSEETAAASAAAPSEASEGTIVVTEGTTIVTQTLGSHGTAVAAQSEAPQGPTVAEQSSVSSSPSTEAVSAYE**G**AGNTVSYSFGTVLMVAAAILL |
|  | *M. guilliermondii*  PGUG_02521 | **MKISVLVAQALVASQFVPGVAA**TWGKTPVTQKTEDCDTTSTYQPTPTYETTPCDTTSTYQTTPPYQTTPCDTTSGSFPTTTKVSVPESDTPATSESTSSKPVTSKSESCSTESDFVPPKSDTTPPKFSTTDCTTTTSESTADTSSTETFPTLCIESTSTSTTVGTTTGSSSTTVVSSVESSTETSVADTSSTRTSTTETPTRETSADSSTVETSTHETSSIETCTTETSVAESSTHGTSTVETSSVETSILGSSSVETSTVESSTIETTSVESSTVETSTVESSTVETSTVKSSTVETSTTETSTTEHSTECSSTRTSVAETSKESSASSTKLSVTSVISTAESSSSVGPHYWTTTETCSTSTIVTVTSCEEQCTEATLTTGVTVVTESEIVHTTYCPLTFTTGVTVITEGETSYTTYCPITQGKEATVVPSGIPTVVPGTYESTVVITITSCEGNKCEPVPVTSVQKTIVTETITGVAPATTQTATEGAKASPIATEASPIPTKAYTPIEASASIQFAAPVPAPASTPVASKAYPATVAGQSTAIESGASSSPIETVAAPSISEQT**G**AAGKAVLSSGAFLVGVAAMLL |
|  | *D. hansenii*  DEHA2G17820g | **MKFSSGVLLSAAAAPALG**ASSAYTNGTSTATDLQTTVVTITSCSDNACSTGVQTTGLTTVTKDETVYTTYCPLTEGESTSAAPAETSPATGGEETKTNIETAVVTITSCSDNACSTGVHTTGVTTITENDTVYTTYCPVSEAPAGTTPASEAPAGTTPAGEAPAGTTPAGEAPAGTTPAGEAPAGSAPAGEAPAGSAPAGEAPAGSAPAGEAPAGTTPAGESTVAAEATTPAGEAPVGTAPAGESTVAGEATTPAGSAPAGQSTVAAQSTSAAESSVAEVSAAE**G**AANNKAVPVFVAGLLAALSLL |
| *Candida albicans* Hwp2 | *C. albicans* Hwp2  C4_03510C_A  orf19.3380 | **MRFATTQLATLACFILTAEA**TFPLRGLFNDAPVDVDLGVYHEESGNNKEQKVDGFNMSPNIKKRTNENNAANVVSTNGGLFITSTKELKTTVVVTSCFNNVCSETSITTPKTAVTATTSKHSTSKPTYTTTSKHSTSHSSTPASTSKHSTSTSTHPATSEHSTSKSTHATSSKHSTSKSSVSVTTSKHSTHDTTSKSFVTPPASSTTSEHTKHKSHKPSKTVVTLTSCSNNACSQSEITTGAIVVTDKETVYTTYCPLTDTETETESTTATTSKHSTHTTTSKHSSVESTSVTSSSKHSVSKSTDVTTSKHSSSESSHATTMKHSTSKHSTHATTSKHSTTESTSGITSKHSTHATSSKYSTVESSSSFASTSESSVPVSSSKSTTFESSISTTTSKHLTLKSSTPASTLEYSTSIPPAPATTSNSLSTKSTTLTTISRSSTSGSSVPNTTRESSTSTTTPNSSSSESKVSSAIPKYSSSEVSSSATTLKSYSTTHSIPTTLVYSSSTSLGFSVTEFRNLTTTSKSSLSTSTTELLTSGTTVRSSTSESSVTSATSIYTSSESTTSSESTTSIETPKSIASKSSSSVTLPKSSTFAWSTSTTTPESSPITLKLSTSKPPKPSATMESSASTTKNSSIQSTSEATTSGSSGVESSVLTATTKSSVPVTTSEWSSVVTTPKSSAPNTTLEHSTSASETSSGSVYTTFDQSTTVITVTSCSDNLCSKTEVTTGVTVITSDTTSYTTYCPLTGTTTVSSALESLVTANKSTSYVGATPIVSSVVSTTPIISSASTTPIISSASTTSVISSASTTSVISNAISNPVSTDVKPTTSSQGTKSTPVDTDSKSTSETTVMVYTTKSVTPTTVESISVAVSSAAQSSIAAISSYE**G**TGNNMKLSFGVVIAGVAAFAI |
|  | *C. dubliniensis*  Cd36_43420 | **MRFATTQLATLAWFILATEA**TIPLKGFFNDTNKNAAPVITELGVYHEEESTNDQQQKVEGFNIDTNIKKKRNNGNSGDFNGEVFITSTKELKTTVVVTSCLNNVCSETSITKPKTTVIATTSKHSTSKPTHATTSKHTTAKSTHATTSKHTTAKSTHATTSKHSTSKPTHATTSKHLSSKSTHATTSKYLSSKPSHATTTSKHSTSKPPFPFTTTKSVTPLSSSIASTKHTNHKSHKPSKTVVTMSSCSNNACSHSEVTTGVIIVTDKETVYTTFCPLTDTDTEIETISKHSTHATTSKHLTHATTTSKYSTSKPPFPFTTTKSVTPLSSSIASTKHTNHKSHKPSKTVVTMSSCSNNACSHSEVTTGVIIVTDKETVYTTFCPLTDTDTETETISKHSTHATTSKHLTHASTTSKHSTSKPPFPFTTTKSVTPLSSSTASTKHTNHKSHKPSKTVVTMSSCSNNACSHSEVTTGVIIVTDKETVYTTFCPLTDTDTEIETISKHSTHATTSKHSTHASTPKHSTGESSHATTVKHSTAKHSTHATPSEHTSVESSHATTIKHSTAKHSTHAIPSEHSTVKHSTHATPSEHTSVESSHATTVKHSTAKHSSHATPSEHTSVESSHVTTIKHSTVKHSTHATPSEHSTHASIPKHSTGESSHATTIKHSTAKHSTHATPSEHTSVESSYVTTVKHSTIKHSTHATPSEHTTVESSHATTIKHSTAKHSTHAIPSEHTSVETASGTTSEHTSVKSSHVTTIKHSTAKHSTHAIPSEHTSVESSHATTVKHSTAKHSTHTTPSEHTSVESSHATTIKHSTAKHSTHAIPSEHSTVKHSTHAIPSEHTSVESSHATTIKHSTAKHSTHAIPSEHTSVESSHATTVKHSTVETALGTTSEHTSVESGSVTKSIHVKTSRQSTVESTSSVASASESSDLVNSSESTAFESSISTTTSKDSTSKPSTSVSTLEYSTSKPPAPATTLEYSTTESTILSSTSKSSTLGSSVPSSAPESSILTTTPDSSSESLISTTTSKYSSSEFSSSPTTLKSYSTKSTIPGTIVYSSSTTLGYSVTKFSNLTTTSKPSLSTTTTELFTSATTLKSLVSESSVPESSIISTTLMYSSFDSTTSIETPKSTISESSGSANPSKSSTSAWSTLTTTPESSPMTSILSTNKSSKPSTTPESAVVTTKNPSTESTTEATTWESSAFESSVSTTTPKSSVAVTTPEWSSVDTTSNPSVPNTILEHSTSAGETSSGSVYTISEQSTTVITVTSCSNNLCSKTEVATGVTIITSDTTSYTTFCPLTETTPVSPALESSVTVGKSTSNIETTPVSESAVSTTSVASSAASTTSVGSSAASTTPVSKSAVSTTSVASSAASTTSVGSSAASTTPVASSAASTTSVGSSAASTTPVSKSAVSTTSVASSAASTSITPDVKATTNSQGTKSTLVGTDSTSTSETTVMVYTTKSVASTTLESISVAVSSAAQSSVAAISTYE**G**TGNNMKFSFSVAIFGIAAFVI |
| Identified by BLAST, not in a CGOB pillar | *C. parapsilosis*  CPAR2_602610, CPAG_02691 | **MLFFITQLMSLALLVVA**TYHETENVRVFKDWATPPLNSVCTVKQLYCDTTTNKKDCGRLTFNSAFVVSDPRKNEDGTFNVVMRYEADHSDKLYEYFAENVASISVAGTGSEDVYIYRKDRFKGIESPFRWSKQIQVKPQIRQGKCCLPDTVSIGYTFANYGPGWSALNCKFGKSCVYRMKPNFKFVETCDAESFFNKQWSKFKRDDELADLEKRTNKYESLLHLCTSKTYKIRQMCWDCDCPPSPTTVTVTPSSAARQSSEKPSIATCSSAVPVMPTPITITTTQVTTIYTLGTTIVSTYCPSTVIYPSTTSSILESSSSTAAVLSSTPVVSSFTAVSLSTAIVSSSAIVSSSAIVSSSAIVSSSTPVASSSAAIVSSSTSIVSQESTSAVVVSTSSKATSITPPTSSPASPSSSSGAFSVSEAIMSTTSSQKSIVPTNPSLVSPSQSSLSASSRQTSAVISSLQFESSDIDVGTTGSIYLSSQSAGFEATSVGTTTRSGAAIDATESGATGASAASSEATSSGATEIGSGSSSATGTNATEFSTGSSSAPSTDSTETGAVVTDAGSMIAISTSGTSTDTDSAGVGSITVGEDSGSTSVSNGGSQPTDASVGSTGDEATTRADITSSAGSTTDIDKTSASGTELIGGPANTPGAESSAGSENTAGTVSNSEPESTPYLNPTSSKGPSSDEGNNNDTGSTSGESDSNGTGSTSGQAGGDYESSGSNDKDGSQLGNDAGDESSSSTTLTTTLVSTMASSGPGHQGGQGGQGGQGNVDAGSGSGGFSGVGDDVVATIETYS**A**AGAAIEYSIATTLIGLAAAILL |
|  | *C. orthopsilosis*  CORT_0E05950 | **MRLSTIQLLVYFVCSIQA**SLVFFPNSFNDCTPPTKPVCTVNEVYCDNNSNNPQYSNANFNAAFVVSEAEKNADGSYNVVGNYQAAQSDQLFNNFAQNVDQIYISGTGVNDATIYSQSTNNPVNNPFKWSAKFRCQPQIKFGKCCIPDGLKLYYKFKQGGAGGAVLNTVFGQQQLSYYNTNPLSGDAQLYDPNNLFNQQLSFHKRDEEADVDQRTELSKRTSNGLLNLLISCTNKHQGIKQFCWDCDCTPPSSSSEPPSSSEPPSSSSEPPSSSEPPSSSEPPSSSEPPSSSEPPSSSSESPSSSEPPSSSEPPSSSSESPSSSEPPSSSEPPSSSEPPSSSEPPSSSSEPPSSSEPPSSSEPPSSSSEPPSSSEPPSSSEPPSSSSEPPSSSEPPSSSEPPSSSSEPPSSSSEPPSSEPPSSSSSEPPSSSEPPSSSEPPSSSEPPSSSEPPSSSEPPSSSEPPSSSEPPSSSEPPSSSEPPSSSEPPSSSEPPSSSEPPSSSEPPSSSEPPSSSEPPSSSSEPPNSSSEPPNSSSEPPSSSSSPPSTTTPPFTAVIETSTIDSTTVITITSCFYGGCSTITETTGLTVITEGTTVYTTYCPLTGATITSTTTGKGPSNGSGSNNGSGHGSGSGSGSGSGSGSGSGSGSGSGSEGNNGSGNGSGNGSGSGSGSGSGSGSGSESGSGAVSSNNNGSGSTTVITVTTCSKGGCSTLTETTGVTVITEGTTIYTTYCPLTGEAPPSSLSIAPSNVSGSGSGSGSGSGSGSGSGSGSSHESGLEGNNGSGNGSGSGTGSGSGSGVASGTTGTGNESNSELATNASQQPNSGVGSGAGSGLSGSEAGGTSPGAASTGTDQESGVGPGQASSVASDSTSAVSNTPQFSPLAGGAAAKGYTIGSMVIAFAMILTLDLSYFKTIFQQLCRRFVFRLCLQVL |
|  | *C. metapsilosis*  Cm_4_904913 | **MRSLNLQLFALLASQAHA**LVFFPNSYTDCSSPTPPPICHVTNFYCDSQPLTQTSVRTIDSAFVISDASKNNDGTYDIVANYQAEYASQLKSDIGSNVDQIYVTGTGVDDSTIFNSKVTNNQVTNPFEWSSTFRCKPQIKNGKCCIPDTFQLWYKFKQGGAGGLAVAGVFGLLLSSYYAVKQISGNVQTYDPNGLFNQQLPPQKRDISELNEVDKRAGNPYLAFVGSCSSHFYGVKQFCWDCDCPPPPSSGTPSSAPSSAPPSSEPSSAPSSEPSSEPSSAPSSEPSSAPSSAPSSEPSSQPSSQPSSEPSSEPSSEPSSAPSSEPSSAPSSEPSSEPSSAPSSEPSSAPSSEPSSEPSSQPSSEPSSAPSSEPSSQPSSEPSSAPSSEPSSQPSSEPSSAPSSEPSSAPSSEPSSQPSSAPSSEPSSAPSSEPSSAPSSEPSSQPSSAPSSEPSSAPSSEPSSQPSSAPSSEPSSAPSSQPSSAPSSEPSSEPSSEPSSAPSSAPSSEPSSAPSSEPSSQPSSAPSSEPSSAPSSEPSSAPSSEPSSQPSSAPSSEPSSAPSSEPSSQPSSAPSSEPSSAPSSEPSSAPSSEPSSQPSSEPSSAPSSAPSSEPSSEPSSQPSSEPSSAPSSEPSSQPSSEPSSAPSSEPSSEPSSQPSSAPSSEPSSEPSSEPSSAPSSAPSSAPSSAPSSEPSSQPSSQPSSEPSSQPSSAPSSAPSSEPSSEPSSAPSSEPSSQPSSQPSSAPPSSGPSSSPPPASSSPSFTQVIETSTVESTTVITTTTCSHGACSTITETTGLTVITEGTTVYTTYCPLTGVTSTSTITGGSGSGNGSGNSAGHGNGNGSGSGSGSGSGSGSGSGSGSGSANGHGSVNNGAGSGSGSGSGSASGSGSGSGSGSGSGSGSGSGSGSGSGSGSGSGSGSGSGSGSGNGSGSGSGSGSGSGSGSGSGSGSGSGSGSGSGSGSGSGSGSGAGNGSGNGNASGHGSGGGSGSTTVITITSCTNGGCSTITKTTGLTVITEGTTVYTTYCPLTGTTTTSTATGGAGGASSHASGSGSGSGSGSNPGSGSGSSPGSGTQSGSGSGSSPGSGSGSGGQSGSGSGGQSGIGSSPGSGSGSSPGSGSRSSPGSGSGSGSGSGSGSGSGPGSGSSAGGNNSPGGSTTTASDGQGAQGAQGAQGLSQAGTGPNQAPAGAALAQPSSVAQQSVLAESTTSSGSNPPQFSPLAGD**G**TSLRLTMGGALAAAVVMLL |

* Putative secretory signal peptides were printed in bold type, Kex 2 cleavage sites underlined, and putative GPI anchor addition sites marked in bold red type.
