# Supplementary material for: Development and Use of a Monoclonal Antibody Specific for the Candida albicans Cell-Surface Protein Hwp1
Source: Front Cell Infect Microbiol. 2022 Jun 27;12:907453. doi: 10.3389/fcimb.2022.907453 (PMC9273023; doi:10.3389/fcimb.2022.907453)
Supplement: Supplementary file 5 [file Table_2.docx]

**SUPPLEMENTARY TABLE S2.** Amino acid sequences corresponding to the entries in **Table 2.**

| **Gene Identifiers and Putative Protein identification/Function** | **Protein Sequence*** |
| --- | --- |
| *C. albicans* Hwp1; C4_03570W_A; orf19.1321 | **MRLSTAQLIAIAYYMLSIGATVPQVDG**QGETEEALIQKRSYDYYQEPCDDYPQQQQQQEPCDYPQQQQQEEPCDYPQQQPQEPCDYPQQPQEPCDYPQQPQEPCDYPQQPQEPCDNPPQPDVPCDNPPQPDVPCDNPPQPDVPCDNPPQPDVPCDNPPQPDQPDDNPPIPNIPTDWIPNIPTDWIPDIPEKPTTPATTPNIPATTTTSESSSSSSSSSSSTTPKTSASTTPESSVPATTPNTSVPTTSSESTTPATSPESSVPVTSGSSILATTSESSSAPATTPNTSVPTTTTEAKSSSTPLTTTTEHDTTVVTVTSCSNSVCTESEVTTGVIVITSKDTIYTTYCPLTETTPVSTAPATETPTGTVSTSTEQSTTVITVTSCSESSCTESEVTTGVVVVTSEETVYTTFCPLTENTPGTDSTPEASIPPMETIPAGSEPSMPAGETSPAVPKSDVPATESAPVPEMTPAGSQPSIPAGETSPAVPKSDVSATESAPAPEMTPAGTETKPAAPKSSAPATEPSPVAPGTESAPAGPGASSSPKSSVLASETSPIAPGAETAPAGSSGAITIPESSAVVSTTEGAIPTTLESVPLMQPSANYSSVAPISTFE**G**AGNNMRLTFGAAIIGIAAFLI |
| *C. dubliniensis* Hwp1; Cd36_43360 | **MKLSTAKLVAIAYYMLSIGATIPSVDD**QQVEEGLIQKRTYGYYQEPCDDYYPPQQQQEEPCDYPQQQPQEPCDNPPQPEEPCDNPPIPNIPTDWIPNIPTDWIPNIPNEPTNPPTTPNIPATTTTSESSSVPSTTPKTSASTTPESSVSATSPYTSVPTTIPKSSTPATGPETSVPGSSILATTSESSSVRATTPNTSVPTTASESSTLETKTSITPLTTSTEHDTTVITVTSCSNSACAESEVTTGVVVVTSEDTIYTTYCPLTETTPGTESTPEASTPIMETIPADSEPSVPASETSAVVPELSVPTTESAPGTKMTPGGLEPSAPVSEMTPAGIKTNQAVPESSIPAGSSVAITTPVNSTIVSTTEGAIPTTLESVPIMQPSANYSSSVAPVSTFE**G**VGNNIRLTYGAAIVGLAALLI |
| *C. albicans* C2_07440C_A; Sterol esterase | MSEENPPQQDQQAPNVSDKPQGDSLITPRETHFHNVFVKYLIMLISALGSVIYTTVLCTGSILNHWFDIVSGREKTVLADIEQRSHGYVPREPYDELSKMKPTSDLQYYLQQLNLDLQEYRVTTCDGYILTLHRIIDPRESEEQRQQRKPVLLQHGLLSCSGTWIVSGKNSSGYYFHEQGYDVWMGNNRSWFIPQHKTLSGSLYNNEQYWDWGVQELAYHDLPALISTVLANKKYFQKLVLLGHSQGGLQSFLMLKNPRLGEIHEKVELFCPLAPAVYPGKLFYTRQFIKFINNRSEFMWLILFGCCAFLRNLCLVRHYIASSWLFGKLSYYMFKYLFGWTGRNWGPYKKVWHFCFIFNMSYASVELMKYYLSKHSDCGFTTLLQPKAAYSSDAHFTENVTDDKKSYFQFDTTWFSGIKVPMIVFIGDEDFLVDGEKVVAHMRKYEPGYREGSNFEAVSIPTYNHLDIVWAEDVIGTIGYTICNKLKQMKARDVQEGLTSESKEQAVTIPEGSSGHHQQQEEVGLNEKIVSVSEDIHDTELTTKVKHNLSADIPLTETRKLTTTEVF |
| *C. dubliniensis* Cd36_54350;  mRNA binding protein Ssd1 | MSSSQDYNNNNNNAPARVSSRKGKNLHVAHRRSPSELTNLMVEQYNLQRQLEEVQAQQKLLEEKQKQQQQQFTYPLAQGSSELAPPPISSGYGGNRSSHSRSSSINTHRRTGSGSGGTAHGHSRRHSLGLNEAIKAAANQKQSNRNSLSPTIPSEVKVDSSDDIGSFKFPPSSGDQGDSSGQSSSHNRSRSLAYGQQSFKFPPTPDQSNDNRGNSLLPPNPNFTVTQSPDRGHNRRSSHFRTASRGSGNNNTDGINSNWRAQQQSPQQQQRSGGLLEPPQVGFTPGHKPRNSSYGGGSSVSSLQQFLPNNGGSNNSSQQGGHQGGGNNGRKTLFAPYLPQSSLPELINEGRLVTGTLRVNKKNRSDAYVSTDGLLDADIFICGSKDRNRALEGDLVAVELLIVDEVWESKKEKEEKKRRKDNTLNSRPLTDDIHNDATSAPNTAEGSVTGTSKEDGAGSNEEETGGLARRGSLKQRPTMKKNDDVEVEGQSLLLVEEEEINDEIKPLYAGHVVAVVDRIPGQLFAGTLGLLRPAQAAQAARDKKNGKESTVQNPKAPKIVWFKPTDKKVPLIAIPTEQAPKDFVENHEKYADRLFVASIKRWPITSLHPFGTLVSNLGPIDSPETEIDSILRDNNFLCDEYPDDDDENDIVSVNAYDLPPIEPEFEGAQREEYLNDYIIAFTQNGEFVDHALHVKRISNTKIELGFHAADIAYFIKPGSSLDRKSKKRSSSVFLPQKTVNLFPKQVNKMVSFKENEKNLAVSVVFEIDTSNFEVEDLYIHESVIVPKQLVTYDAFDTILSGQSVDSISSATSDYVKTFSLIAKEFRRHRLSNRSLGITPNLTLLDQLDDEKVRLDLNIFKDSLAFDVISEISHKVNSAIAAKVHAGLGDQAILRRHPLPTLQKMETFVRKATSLGFKIDTTTSSTLQNSILKIDDPVKRKCVETLLYKCMSRGKYYVAGKQDADSYAHYYFNLPLYTHFTAPLRRYADLIVHRQLKAVLNKQVEDKDLDSLKAITDYCNFKKDCAASAQEQAIHLLLSQTINEMSETAGQLLCMGTVVQVYESSFDVFIPEFGVEKRVHGDQLPLVKAEFDKGERILELWWEKGVDSATYIPPDEKSSLSYRNSIKNKYRTSALQAAKIQSKTALEKSTTPADSVAEKLSKLNLEPPKLTVPSLKGNELNAVEKDETRSMPSSPTQAEIPKNIRTNSSSRISASGNTFSLEPYLQNTITRVEGDSYIQVIKELTQVPVLLRAEIGMALPCLTVRVLNPFAEER |
| *M. guilliermondii* PGUG_05369;  Phosphatidylinositol 3,5-bisphosphate-binding protein | MNTRSPITGDDSETNVLDIAFNQDQGCFAIAHEHGFLVFNTDPLDLRVKRKFAGTSSGVGHIAMLHRTNYLALVGGGINPRYPETKLMIWDDLKRKNSLQLDFSTPVLNVLLSRTRIVVVLKNHVHVYGFSSQPHKIASYETSDNQHGLADLSVNVSYNDIDSSASTSGESSNSNDSKHEGKQQTLAFPARTAGQIHLVDVSTQGQERNVVNIIKAHKSSIRCLTLNRSGTLIASASETGTIIRIHSTRSTALLFEFRRGLDRADITSMRFSRDDSKLGVLSDKTTLHVFNINPSQQEQPDDEVKAPTNRHHLFSFLPVPVPTYFRSVWSFCSVNTNSDHPRSEENDTGVIGWSSNDSIIVVWKKKQLWERYVIGQAANGWQLTRHSWRSLAIEDVDKW |
| *M. guilliermondii* PGUG_03203; Ribitol kinase | MGKSNLNLSSRKSSLFFQQNPPQPDVYYVGVDVGTGSARACVIDTNGIILGLSERPITRHELKPNYITQNSTEIWNAICFCVKRAISESGVDPADIFGIGFDATCSLVVLRESTDEPVGVGPDFTDNHENIILWMDHRAVDETNTINATGDKCLKYVGGQMSIEMELPKMKWLKHNLPGGIDDCKFYDLADFLTHRATGSEARSFCSTVCKQGFVPLGVDGSETGWSKEFLESLDLPELVEDNFRRLGGSPASGATWLSAGDIVGKLNSQAADELGLTTECIVGSGVIDAYAGWIGTVAAKADIPSLSDSKEEGIAISCGRLAAVAGTSTCHIAMTKEPCFVNGVWGPYKDVMAPGYWCAEGGQSCTGALLAHVLAIHPATTELSRLSEASNLSKFDYLNLTLETMVSETKSRSVVSLAKHMFFYGDFHGNRSPVADPRMKASIIGQSMDTSVQDLAIQYFGACEFIAQQTRQIIEEMENSGHKIDCVFMSGGQCRNGLLMRLLADCTGLPIVIPRYIDAAVVFGSALLGAVASEDAVLEHIHDRKRSRRSSILSNQKSQSNLTNIDGPHSPYTAPTATASSTNMTALAYASTQSGSHHQFPTMTPMAEETDYFNQATQSKKEEESDSDDEQTLSFGSKQNVQQGLSQKLERMGIKPLSAADKKDKKDGKPGDKLWKIMERMTGPGRVITPSSPTHPDRKLLAAKYKIFLEQCKTQRTYRDMVDEVEKENLAAK |
| *D. hansenii* DEHA2A06886g;  Aromatic amino acid aminotransferase | MLNKLLNGVIPPFNFCHNLDRYYDRRFDNAVKVLTCTFSGDFRILQVMYLLLIIYYIRTYILLYFIICNKHIDSTKKDMSKPEAKDLSHLLSEEAKSRKNSPLKAAFKYYKQPGMTFLGGGLPLSDCFPFNKVTADIPSVPFSEGISAPLSDENKTVVEVYKRAEMNKTEDKQIELARSLQYGHTEGQAEIIDFLKEHTEKIHNVPYKDWDLIASVGNTQSWDATLRSFVTRGDSILVEEYSFSSALETAHAQGVNTIPVPMDAKGILPEALEKQLDQWVGPKPKLLYTICTGQNPTGSCLSAERRRAIYKLACKHDFIIIEDEPYYFLQMEDYTTDEKSRASNKVDSHDEFVKALVPSYISMDVEGRVIRLDSFSKVLAPGLRFGWIVGQAKLLERYVRIHEVSIQCPSGFTQSICNSLLQKWGQKGYLDWLMGLRADYTHKRDVAIDAVNKYFPKEVVSFIPPVAGMFFTTTIDASKHPKFKSEFNEDPLQVENAVYEQALKQGCLMIPGSWFKADGKSNPPQPELPENPASKNTFFFRGTYAAVPLDQLVIGLEKFGKAIKVEYGL |

*Kex2 cleavage sites were underlined in the *C. albicans* and *C. dubliniensis* Hwp1 sequences. Putative secretory signal peptides were shown in bold type; putative GPI anchor addition sites were marked in bold red type. These features were only present in the Hwp1 orthologs. Sequences with identity to the anti-Hwp1 immunogen peptide were highlighted in yellow.
